# Matrix Metalloproteinases and Their Inhibitors in Pulmonary Fibrosis: EMMPRIN/CD147 Comes into Play

**DOI:** 10.3390/ijms23136894

**Published:** 2022-06-21

**Authors:** Lourdes Chuliá-Peris, Cristina Carreres-Rey, Marta Gabasa, Jordi Alcaraz, Julián Carretero, Javier Pereda

**Affiliations:** 1 Department of Physiology, Faculty of Pharmacy, University of Valencia, 46100 Burjassot, Spain; lourdes.chulia@uv.es (L.C.-P.); cristina.carreres@uv.es (C.C.-R.); julian.carretero@uv.es (J.C.); 2 Unit of Biophysics and Bioengineering, Department of Biomedicine, School of Medicine and Health Sciences, University of Barcelona, 08036 Barcelona, Spain; aeryn13@gmail.com (M.G.); jalcaraz@ub.edu (J.A.); 3 Thoracic Oncology Unit, Hospital Clinic Barcelona, 08036 Barcelona, Spain; 4 Institute for Bioengineering of Catalonia (IBEC), The Barcelona Institute for Science and Technology (BIST), 08028 Barcelona, Spain

**Keywords:** pulmonary fibrosis, MMPs, EMMPRIN, CD147, basigin, TIMPs

## Abstract

Pulmonary fibrosis (PF) is characterized by aberrant extracellular matrix (ECM) deposition, activation of fibroblasts to myofibroblasts and parenchymal disorganization, which have an impact on the biomechanical traits of the lung. In this context, the balance between matrix metalloproteinases (MMPs) and their tissue inhibitors of metalloproteinases (TIMPs) is lost. Interestingly, several MMPs are overexpressed during PF and exhibit a clear profibrotic role (MMP-2, -3, -8, -11, -12 and -28), but a few are antifibrotic (MMP-19), have both profibrotic and antifibrotic capacity (MMP7), or execute an unclear (MMP-1, -9, -10, -13, -14) or unknown function. TIMPs are also overexpressed in PF; hence, the modulation and function of MMPs and TIMP are more complex than expected. EMMPRIN/CD147 (also known as basigin) is a transmembrane glycoprotein from the immunoglobulin superfamily (IgSF) that was first described to induce MMP activity in fibroblasts. It also interacts with other molecules to execute non-related MMP aactions well-described in cancer progression, migration, and invasion. Emerging evidence strongly suggests that CD147 plays a key role in PF not only by MMP induction but also by stimulating fibroblast myofibroblast transition. In this review, we study the structure and function of MMPs, TIMPs and CD147 in PF and their complex crosstalk between them.

## 1. Introduction

In lungs, interstitial space is almost absent to minimize the distance between blood and the alveolar cavities. Physiologically, fibroblasts and other connective cells such as leucocytes reside in this microenvironment in a latent state. As a consequence of reactive or regenerative processes, fibrogenesis occurs to produce wound healing and to replace or fill the gaps generated by the disorders of the parenchymal tissue. Typically, fibroblasts proliferate and transform into myofibroblast-like phenotypes characterized by the expression of alpha-smooth muscle actin (α-SMA) and secretion of extracellular matrix proteins. Several stimuli may trigger those processes, such as pneumocyte apoptosis, repetitive inflammatory responses or other parenchymal disorders. In pathological situations, fibrotic responses become chronic, causing the enlargement of the interstitial space, increasing the distance between blood and alveoli and replacing air content with cells and/or ECM. Other cells such as macrophages or smooth muscle cells will cooperate or accumulate causing a disorganized structure. Thus, diffusion and ventilation are impaired and respiratory failure occurs. 

The most characteristic fibrotic lung disease is idiopathic pulmonary fibrosis (IPF). This chronic, progressive and fatal disease of unknown cause typically affects elderly adults (>60 years). The incidence of IPF was estimated to range from 0.2 to 93.7 per 100,000 inhabitants, with values of 3 to 9 per 100,000 inhabitants per year in Europe and North America, generally higher in men than in women [1]. Usual interstitial pneumonia (UIP) is the histopathological pattern of IPF. It is characterized by a combination of patchy interstitial fibrosis with alternating areas of normal lung, ECM deposition, presence of fibroblast foci and parenchymal disorganization due to chronic scarring or honeycomb change. In this context, cells producing and remodeling ECM play a key role. Myofibroblast phenotype emerges because of the fibrotic response. At the same time, myofibroblasts are considered as effectors of fibrosis due to their capacity for secreting and remodeling ECM. In this sense, myofibroblasts play a central role in the fibrotic feedback loop that occurs during IPF.

## 2. Extracellular Matrix in Pulmonary Fibrosis 

ECM homeostasis arises through the exquisite balance between the expression of ECM components and the expression and/or activation of ECM proteases such as MMPs and their inhibitors such as tissue inhibitors of metalloproteinases (TIMPs) [2]. In IPF, ECM homeostasis is dysregulated through processes that are not fully understood, including the aberrant expression of ECM proteins that ultimately elicits an excessive accumulation of fibrillar collagens (type I and III) [3]. Activated fibroblasts or myofibroblasts are the key cell type involved in the excessive accumulation of fibrillar collagens. Among the different fibroblast activator cytokines, transforming growth factor beta 1 (TGF-β1) is the most potent and well-studied, and it is frequently upregulated in IPF. Notably, we and others have shown that TGF-β1 downregulates the mRNA levels of major collagenases such as *mmp1* while upregulating those of *timp1* and *col1a1* and *col3a1*, which clearly favors collagen deposition. In normal conditions, other signaling factors counterbalance this procollagenous transcriptional program [4]. In contrast, this program becomes amplified in IPF and other fibrotic processes through mechanisms that remain undefined.

Although most myofibroblasts are thought to arise from the recruitment and expansion of resident fibroblasts, there is strong evidence suggesting that bone-marrow-derived cells such as fibrocytes are recruited at injury sites and may contribute to the pathologic increase in myofibroblasts. In addition, endothelial and epithelial cells may contribute to the myofibroblast population through endothelial-to-mesenchymal (EndMT) and epithelial-to-mesenchymal (EMT) transition, respectively [5]. Although evidence supporting these contributions is scarce, we previously reported that lung epithelial cells undergoing EMT in vitro failed to match any biochemical and biophysical marker of primary pulmonary fibroblasts, let alone myofibroblasts. To illustrate this difference, the expression of fibrillar collagens was several orders of magnitude lower in cells undergoing EMT compared to fibroblasts [6].

The aberrant ECM remodeling in IPF not only changes the biochemical microenvironment, but profoundly alters the mechanical and diffusive properties of the fibrotic pulmonary tissue, which may impact both disease progression and response to therapies [5]. From a mechanical standpoint, normal lungs are soft and elastic to allow the cyclic volume changes required for breathing, and pulmonary tissue exhibits a Young’s Modulus (indicative of resistance to deformation) E ~1 kPa. In contrast, tissue rigidity markedly increases in both IPF patients and in bleomycin in vivo murine models of PF, owing to the extracellular accumulation of fibrillar collagens and to the aberrant abundance of hypercontractile myofibroblasts, eliciting an increase in E that can be locally as high as 30–50 kPa [4]. Of note, tissue stiffening alone has major profibrotic effects, as revealed by in vitro studies using culture substrata with tunable elasticity. These studies showed that increasing matrix rigidity up to fibrotic-like values was sufficient to promote fibroblast proliferation and survival through β1 integrin upregulation and subsequent FAK activation [7], and to increase resistance to apoptosis through mitochondrial priming [8], which is a pathologic hallmark of fibrotic myofibroblasts. Similar studies showed that matrix stiffening synergizes with TGF-β1 to promote mRNA expression of both *col1a1* and *mmp1* in IPF-fibroblasts, whereas TGF-β1 and matrix rigidity downregulate *mmp1* in normal pulmonary fibroblasts [9]. The increased deposition of fibrillar collagens can hinder effective molecular diffusion [10], which may impair efficient drug delivery and ultimately limit therapeutic responses.

## 3. Matrix Metalloproteinases—Role in Pulmonary Fibrosis

### 3.1. Introduction

MMPs are zinc-dependent endopeptidases that belong to the metzincins superfamily of proteases and contribute critically to ECM homeostasis. MMPs are best known for their role in degrading ECM components, such as collagens, fibronectins, laminin and others. Their protease activity cleaves or releases from ECM other important proteins such as growth factors, inflammatory mediators and receptors, modulating apoptosis, proliferation, angiogenesis, immune response and tissue repair [11]. They facilitate cancer invasion and metastasis through ECM degradation and cell to cell and cell to ECM adhesion cleavage. All those functions belong to the main pathologic mechanisms involved in PF. Therefore, they play a major role in the progression of the disease.

### 3.2. Structure and Classification of MMPs

Since their discovery in 1962 in the tail of a tadpole during its metamorphosis [12], 23 MMPs have been identified in humans to date, encoded by 24 genes (two genes for *mmp23*). Mice contain one single copy of *mmp23*, two gene encoding paralogues homologous to human MMP-1 (*mmp1a* and *mmp1b*) and lack an *mmp26* gene, comprising 23 genes in total [13]. Similar numbers of MMP-encoding genes have been characterized in other vertebrate models (e.g., 25 in zebrafish) [14].

All MMPs share a common catalytic metalloproteinase domain (about 170 amino acids) containing a highly conserved Zn^2+^-binding motif, HEXXHXXGXXH. The three histidines (H) bind to a Zn^2+^ ion and, together with the nucleophilic glutamate (E), attack the substrate’s peptide bond. Another highly conserved Met-turn sequence (XBMX) assists in the catalytic activity [15].

After removal of the N-terminal secretory signal during translation, the N-terminal domain of MMPs is an auto-inhibitory pro-peptide (about 80 amino acids) containing a highly conserved cysteine switch motif, PRCGXPD. Its cysteine sulfhydryl chelates the active site for Zn^2+^ in the proMMP zymogen form. The cleavage of this domain by peptidases or other MMPs will detach the propeptide domain to release the catalytic site, transforming proMMPs into active MMPs. Between the catalytic and propeptide domains, a furin-like proprotein convertase recognition sequence is found in furin-containing MMPs (e.g., MMP-11, -21 and -28) This sequence allows their intracellular activation by furin-like proconvertase enzymes. At the C-terminal flank, a flexible linker region (or hinge region of variable length) followed by a hemopexin-like domain (about 200 amino acids) is found, which typically is responsible for substrate recognition and dimerization [16]. The absence of a hemopexin domain will have an impact on their substrate affinity in vitro. In addition, membrane-type MMPs have either a C-terminal GPI anchor, or a single transmembrane helix followed by a short cytoplasmic tail.

Similarities between MMPs make the design of specific inhibitors very difficult. Generally, inhibitors are designed to target the differences found on the S_1_’ site, a well-defined but weakly conserved hydrophobic pocket of variable depth adjacent to the zinc-binding site [17,18]. MMPs possess six pockets (S_1_, S_2_, S_3_, S_1_′, S_2_′, and S_3_′), but S_1_ is the most important for substrate specificity [19]. 

Thus, substrate affinity and molecular structure represent the common basis for their classification (Table 1) [11,20]. However, substrate affinity classification is based only on in vitro data, with seldom predicted in vivo functions. Moreover, the range of substrates and MMPs is limited, excluding some MMPs from this scheme. 

These limitations have led to the alternative classification focused on their domain organization. This classification comprises archetypal MMPs (MMP-1, -3, -8, -10, -12, -13, -19, -20 and -27), matrilysins (MMPs -7 and -26), gelatinases (MMP-2 and -9) and furin-activable MMPs (all MT-MMPs, MMP-11, -21, and -28) [16].

### 3.3. Regulation of MMPs and TIMPs

MMPs activity is tightly regulated at multiple levels by modulation of transcriptional, post-transcriptional and post-translational processes. The promoters of genes encoding MMPs harbor several *cis*-elements allowing a different set of trans-activators including AP-1, PEA3, Sp-I, β-catenin/Tcf-4 and NF-κB (reviewed in [64]). Some MMPs are coexpressed, but also functionally related MMPs such as gelatinases or collagenases exhibit different promoters. The presence of AP-1 in most MMP promoters either alone or in cooperation with PEA3 renders these genes responsive to a large variety of cytokines or growth factors, including interleukins such as IL-1β, interferons, EGF, KGF, NGF, HGF, bFGF, VEGF, PDGF, TNF-α and TGF-β1, which is key in the development of fibrosis. In addition, ECM proteins also promote signaling pathways converging in MMP expression through AP-1 and/or PEA3 *cis*-element [64]. 

Like other proteins, their final translation also depends on post-transcriptional modifications and mRNA stability. Some reports have described that mRNA stability plays an important role in the regulation of collagenase MMP-1 and gelatinases MMP-2 and MMP-9 [65,66]. Several MicroRNAs (miRNAs) have been identified to downregulate MMP expression in different scenarios such as *miRNA-202-3p* in IL-1β-induced MMP-1 expression [67], mirna-140 in IL-1β-induced MMP-13 [68], *miRNA-377*, *miRNA-382*, *miRNA-410* and *miRNA-192-5p* in MMP-16 expression [69,70] and *miRNA-211* in MMP-9 expression [71]. The next level of regulation is the post-translational transformation from pre-proMMPs to their active form. Firstly, the signal peptide is removed, releasing the zymogen proMMP. Secondly, at some point after translation, either before or after secretion into the extracellular milieu, the propeptide is removed, releasing the inhibitory effect of the cysteine switch, yielding an active protease. This activating post-translational modification is performed by serine proteases, MMPs or other proteolytic enzymes. MMPs containing a furin cleavage motif are activated intracellularly by furin. In vitro studies reveal that the thiol of the cysteine switch motif can also be chemically modified by ROS, relieving the inhibition of proteolytic activity and yielding a ‘full length’ active MMP [72]. Moreover, despite their secretory signal, many MMPs are not fully secreted extracellularly and may be detected intracellularly in a variety of mammalian cell types. In general, the likely conserved and possibly ancestral intracellular role for MMPs is yet unknown. For example, MMP-2 accumulates intracellularly in mammalian cells due to an inefficient recognition of its N-terminal secretory signal [73].

The study of MMP intracellular trafficking (recently reviewed in [74]) is essential for understanding when and where the function of most MMPs will take place. MMPs, like other cargo, are transported intracellularly via vesicular transport along microtubules and actin filaments (F-actin) [75,76]. Microtubules and microtubule-based motor proteins such as kinesins, as well as vesicle regulators such as SNARE proteins or RabGTPases, have emerged as key regulators of MMP intracellular trafficking. In addition, podosomes and invadopodia, which constitute the invadosome cell adhesions subgroup, are characterized by their abilities to adhere to, and degrade ECM because they are sites at which proteases, including MMPs, accumulate on cell surfaces [77]. Membrane-type MMPs execute their activity bound to the membrane of the cells by a GPI anchor or by transmembrane domains. Moreover, other non-membrane-type MMPs such as MMP-2, -8 and -9 also bind to membrane proteins and exert their functions over the cell membrane [78,79]. Several secreted MMPs diffuse and exert proteolytic activity at significant distances from the cell secreting them. In addition to regulation by proteolytic activation (i.e., removal of the auto-inhibitor pro-domain) these proteases are regulated by endogenous inhibitors, primarily TIMPs and α_2_-macroglobin [80]. 

Finally, MMPs are also subject to endocytosis, which can be followed by storage in endolysosomal vesicles, degradation in mature lysosomes, or recycling back to the cell surface. MMP intracellular trafficking regulation, site-directed release at the cell surface, and MMP recycling allow cells to flexibly reroute this portion of the proteolytic machinery in response to changing environmental conditions and for cell–cell communication [74]. Despite recent advances in post-translational modulation of MMPs, there is still a lack of knowledge in the field that needs to be addressed in the future.

TIMPs are a four-member family (TIMP 1–4) of endogenous protein inhibitors of MMPs. They also inhibit other proteases such as disintegrin and metalloproteinases (ADAMs) and ADAMs with thrombospondin motifs (ADAMTSs) closely related to MMPs. They are relatively small proteins (184–194 amino acids, ≈21 kDa) whose structure comprises an N-terminal domain with inhibitory activity and a C- subdomain that interacts with the hemopexin domain of certain MMPs [81]. They are mainly secreted peptides but may be associated with membrane-bound proteins. TIMP-3 exerts its function adsorbed to ECM sequestered to heparan-sulfate-containing proteoglycans and possibly chondroitin-sulfate-containing proteoglycans [82]. In general, TIMP members can inhibit all MMPs, but they exhibit different inhibitory profiles [83]. For example, TIMP-1 is a strong inhibitor of many MMPs except for most MT-MMPs. They inhibit MMPs reversibly by forming 1:1 complexes, but also play a role in the activation and uptake of MMPs from the extracellular environment. In fact, TIMPs can form non-inhibitory complexes by binding to MMPs but keeping both proteolytic and inhibitory activities intact [84]. For example, the hemopexin domain from progelatinases MMP-2 and MMP-9 interact with the c-subdomain of TIMPs to form a non-inhibitory complex that in turn binds to the active site of the MT1-MMP molecule on the cell membrane. Another “free” MT1-MMP will be facilitated to cleave the ternary progelatinase/TIMP/MT1-MMP complex to activate the zymogen around the cell surface [11]. Additionally, it has been seen in a yeast two-hybrid assays specific interactions between N- and C-terminal domains of paralogues of TIMP-4 with two paralogues of MMP-11. During early myotendinous junction remodeling in vivo, TIMP-4 modulates, but does not inhibit, MMP-11a activity. However, canonical inhibitory interactions are detected between the catalytic domain of MMP-11b and the N-terminal domains of both TIMP-4 paralogues [85].

### 3.4. Role of MMPs and TIMPs in Pulmonary Fibrosis

It was first believed that the balance between MMPs and TIMPs would determine the final accumulation or degradation of ECM. In the case of PF, we may expect a net decrease in MMPs accompanied by an increase in TIMPs, since the aberrant accumulation of ECM is a typical fibrotic feature. However, the diverse pattern of secretion and activation of MMPs found in pathological conditions, such as PF, and their intricated interactions with TIMPs in different locations in vivo show a more complex scenario. Interestingly, most MMPs are upregulated in PF patients or PF murine models.

To unravel these issues, studies of MMP deficient mice in PF models have shown that MMPs promote, rather than inhibit, pulmonary fibrotic responses to injury and have discovered a variety of pathways involved. These mechanisms include MMPs: (1) promoting epithelial-to-mesenchymal transition (EMT); (2) promoting abnormal epithelial cell migration and other aberrant repair processes (MMP-3 and MMP-9); (3) increasing lung levels or activity of profibrotic mediators or decreasing lung levels or activity of antifibrotic mediators (MMP-3, MMP-7 and MMP-8); and (4) inducing lung macrophage phenotype switching from M1 to M2 types (MMP-10 and MMP-8). In addition, other animal studies have demonstrated that MMP-13 and MMP-19 have antifibrotic properties, while MMP-1 and MMP-10 have the capacity to inhibit fibrotic responses to damage [20]. These findings explain the presence of high levels and activity of certain MMPs in IPF that have been named the “fibrosis paradox”, since both aberrant accumulation of ECM and increased protease activity coexist in the disease. Early MMPs studies were focused on ECM metabolism, overlooking other important functions of MMPs related to inflammation, immunity and cell death, which play a key role in fibrosis. Of note, most MMP studies have been performed in vitro, which necessarily removes many potential extracellular protein–protein interactions and other components of the in vivo system. Thus, some MMPs will exhibit antifibrotic functions and other profibrotic functions in vivo (Table 1), but clearly, their role in the disease is far more complex than ECM metabolism (reviewed in [86]). In addition, because lung remodeling in IPF is a dynamic biopathological process, it is unknown whether MMP activity varies over time.

In this context, CD147/EMMPRIN has been initially described as an inducer of MMPs, increasing their function in health and disease. Indeed, CD147 modulates MMPs expression to enhance their functions in PF. However, our current understanding reveals that its function is far more important to MMP induction. In this section, we first describe and analyze the most important MMPs and TIMPs in PF, including the mechanisms involved in their fibrotic or antifibrotic functions. Next, we describe CD147’s structure, expression, and function, to discuss its role in this disease.

#### 3.4.1. MMP-1

MMP-1 (collagenase 1 or fibroblast collagenase in humans) can degrade type I-III collagens in vitro. MMP-1 is expressed in fibroblasts, endothelial cells, bronchial epithelial cells (BECs) and macrophages. Its hemopexin-like domain mediates substrate specificity and interactions with endogenous inhibitors [87]. In addition, the α2β1-integrin pathway is a positive regulator of MMP-1 and increases MMP-1 expression through the binding to it on cell surfaces [88,89]. Interactions between a serine proteinase (urokinase-type plasminogen activator) and MMP-3 activate proMMP-1 [90]. MMP-1 is increased in plasma, serum, bronchiolar lavage (BAL) and lung tissue of IPF patients [91,92] and it is significantly overexpressed in IPF compared to normal lung tissue, according to transcriptional and immunohistochemical data [93]. In turn, there is a bidirectional correlation with hypoxia-inducible factor 1alpha (HIF1α) in alveolar epithelial cells (AECs), which represses mitochondrial oxygen consumption [94] and this factor has been demonstrated previously to be increased in AECs of IPF patients [95]. Transcription of MMP-1 is increased by a single-nucleotide polymorphism in the promoter region at the AP-1 binding motif and it has been associated with IPF [22]. MMP-1 expression in IPF is significantly increased in dysplastic epithelial cells overlying fibrotic interstitium [22].

Recently, using bioinformatic methods, it has been demonstrated that *mmp1* is one of the top three differentially expressed genes (DEGs) with the highest significant up-regulation of human IPF and plays an important role in its development. MAPK (mitogen-activated protein kinase) signaling pathway was the most significant enrichment pathway implicated in the regulation and activation of MMP-1 activity. [21]. In addition, weighted correlation network analysis (WGCNA) of multiple gene expression datasets from the Gene Expression Omnibus database by protein–protein interaction (PPI) network identified increased expression levels of *mmp1* and *mmp7*. This finding was validated in experimental an PF murine model using qRT-PCR and in the GSE10667 dataset [23]. Many other studies have demonstrated that *mmp1* gene expression is up-regulated in IPF in comparison to normal lung tissues [96,97,98]. More concretely, IHC IPF tissue studies have shown that MMP-1 expression was increased mainly in epithelial cells, macrophages [93,98] and stromal cells [96,97,99].

We have found that IL-1β downregulates *col1a1* mRNA levels while increasing mRNA levels of *mmp1* and *mmp2* (the major collagenolytic enzymes), favoring a reduction in type I collagen in human pulmonary fibroblasts. These observations reveal that IL-1β may reduce local tissue rigidity and provide innate antifibrotic protection that could be important during the early stages of fibrotic processes and lung repair [24]. Other studies have demonstrated that the pan-RTK (receptor tyrosine kinase) inhibitor nintedanib can reduce the mRNA expression for *mmp1, 4, 13*, and *14* and pirfenidone is able to reduce mRNA expression for *mmp3* and *13* [100]. In both cell culture and mouse models of large cell lung carcinoma cell lines (LCC), the overexpression of *mmp1* has been described to be necessary for the induction of fibroblast senescence and consequent tumor promotion [101].

For all of this, the overexpression of MMP-1 in IPF is a good example of the above-mentioned MMPs paradox, because MMP-1 can degrade fibrillar collagens, the typical excessively accumulated ECM molecules in IPF [102]. Furthermore, diseases characterized by excessive ECM degradation, such as rheumatoid arthritis and pulmonary emphysema, have been linked to MMP-1 [103,104,105]. A possible explanation for this intriguingly finding is that MMP-1 is largely found in the reactive alveolar epithelium in IPF lungs, while it is essentially nonexistent in fibroblasts in the interstitial compartment, where collagens are deposited [106].

#### 3.4.2. MMP-2

MMP-2 (or gelatinase A) is constitutively expressed by BECs, AECs, fibroblasts and fibrocytes. Even though in vivo activation of all MMPs is poorly understood, in vitro studies reveal that proMMP-2 is activated by forming ternary complexes with members of the MT-MMP subfamily and TIMP-2 [107,108,109]. This activation is performed on the surfaces of fibroblasts and macrophages. Both gelatinases, MMP-2 and MMP-9 (gelatinase B) have an important function in the degradation of ECM components, because they degrade gelatin and the principal constituent of the Basement Membrane (BM), collagen type IV [110,111,112]. *mmp2*-null mice model has not been studied in models of PF so far. However, MMP-2 has the potential to contribute to and favor IPF pathogenesis by degrading lung ECM proteins, inducing EMT and regulation of Wnt/β-catenin signaling [25,28,113].

MMP-2 is overexpressed in IPF lung, close to the fibroblast foci, mainly by reactive epithelial cells and myofibroblasts [93,107]. MMP-2 is increased in BAL and lung tissues of IPF patients [36,93]. MMP-2 is found near epithelial basement membrane breaks in IPF lungs and has been connected to basement membrane degradation [114], which increases lung fibroproliferative responses [115] by inducing angiogenesis [116,117]. Endothelial-derived MMP-2 is associated with fibrotic responses. The endothelial release of MMP-2 [118] is induced by vascular endothelial growth factor (VEGF), an important profibrotic and angiogenic growth factor, contributing to bleomycin-mediated PF in mice [119]. Surprisingly, transcriptional reduction in *mmp2* by small interfering RNA (siRNA) reduces VEGF overexpression [120]. In L929 fibroblasts, chemokine (C-X-C motif) ligand 14 stimulates *mmp2* overexpression by downregulating protein phosphatase magnesium-dependent 1A (PPM1A), resulting in the fibrotic response [121].

It has been reported that MMP-2 plays an important role in EMT. It causes an abnormal stimulation of the Wnt/β-catenin-signaling pathway, which influences the pathophysiology and progression of IPF [25,122]. The suppression of β-catenin by siRNA slows the progression of fibrosis and lowers the levels of pulmonary TGF-β1 and MMP-2 by Western blotting [28,113]. A higher baseline level of MMP-2 in old mice’s lung tissue than in younger mice’s lung tissue shows that the aged lung has a profibrotic character that makes it more vulnerable to injury and, hence, raises the probability of IPF development [27]. TGF-β1 activity is inhibited by the secretory leukocyte protease inhibitor (SLPI), which has a protease inhibitory site at leucine 72 in the C-terminal domain. Although SLPI null animals with overexpressed MMP-2 expression show longer wound recovery and enhanced localized scarring, they do not develop PF [123]. The tumor suppressor phosphatase and tensin homolog deleted on chromosome 10 (PTEN) inhibits the expression levels of several crucial proteins in TGF-β1-induced fibrosis, including MMP-2, MMP-9 and α-SMA by blocking the phosphatidylinositol 3-kinase/Akt and TGF-β1/SMAD3 pathways, reducing fibroblast-to-myofibroblast transition (FMT) [124].

#### 3.4.3. MMP-3

MMP-3 (or stromelysin 1) is mainly expressed by BECs, AECs, alveolar macrophages (AM) and fibroblasts from PF patients and can degrade type IV collagen and basement membrane proteins in vitro. MMP-3 expression is increased in serum, BAL and lung tissues of these patients [29,125]. It has been described that MMP-3 promotes PF by three different mechanisms: (1) MMP-3 can activate Wnt-β-catenin signaling in type II AECs by increasing CDH1 (E-cadherin) breakdown and inducing EMT in lung epithelial cells [29,125]. When *mmp3*-null mice are exposed to bleomycin, the expression of cyclin D1 (a Wnt-β-catenin pathway target gene) is reduced compared to wild-type (WT) mice [29,126]; (2) MMP-3 promotes fibrosis by activating TGF-β1 from its inactive latent form to its active form by stimulating the TGF-β1 homodimer to release from latency-associated peptide (LAP) and latent TGF-β1-binding protein-1 (LTBP1) [30]. This produces a reduction in antifibrotic mediators and an increased level or activity of profibrotic mediators in the lung; and (3) MMP-3 causes the inhibition of distal epithelial repair by supporting the bond between endostatin and type XVIII collagen (a proteoglycan found in alveolar capillary and epithelial basement membranes) [31], allowing endostatin to promote lung epithelial cell apoptosis. Concretely, endostatin levels are higher in IPF plasma and bronchoalveolar lavage fluid (BALF) samples, and they have an inverse relationship with lung function [127]. For this, MMP-3 can promote aberrant epithelial cell migration and other abnormal repair processes as the induction of myofibroblast differentiation of fibroblasts and degradation of ECM components.

Bleomycin-induced fibrosis mouse model shows an increase in MMP-3 lung levels in comparison with control and *mmp3*-null mice protected from bleomycin. Yamashita et al. demonstrated that in the rat lung, transient adenoviral vector-mediated production of recombinant MMP-3 resulted in myofibroblast accumulation and PF. On the contrary, *mmp3*-null mice were protected from bleomycin-induced lung fibrosis [29]. Recent studies have described that pirfenidone, a drug approved for IPF treatment reduces mRNA expression of *mmp3* in a profibrotic model of human lung fibroblasts stimulated by TGF-β1 [100]. In conclusion, it seems to be clear that MMP-3 has an important profibrotic function in the IPF context.

#### 3.4.4. MMP-7

MMP-7 (or matrilysin) is expressed by blood monocytes, AM, BECs, AECs and fibrocytes and is increased in plasma, serum BAL and lung tissues from PF patients. Extracellular matrix components such as type IV collagen, laminin, elastin, fibronectin, gelatin and osteopontin (a multifunctional cytokine that regulates cellular migration and adhesion) have a high substrate affinity for MMP-7. MMP-7 can also activate proteases such as proMMP-1, proMMP-2 and proMMP-9, as well as process several bioactive substrates [128]. Like *mmp3*-null mice, *mmp7*-null mice are protected from bleomycin-induced fibrosis [98]. In vitro experiments show bidirectional correlation with osteoponin [91,129,130]. In addition, MMP-7 is expressed by macrophages and airway epithelial cells in IPF lungs. In injured lung epithelial cells, MMP-7 acts as a sheddase for syndecan-1. It can transport CXCL1 as cargo on glycosaminoglycan chains, resulting in the release of syndecan-1-CXCL1 complexes, which are required for neutrophil transepithelial infiltration. Through this mechanism, MMP-7 regulates chemokine mobilization and transepithelial efflux of neutrophils in acute lung injury [131]. Other experiments have demonstrated that it may regulate pulmonary localization of dendritic cells then express CD103 and favors the cleavage of CDH1, which could activate CD103 dendritic cells to limit inflammation and inhibit fibrosis [132]. Moreover, by proteolytically cleaving ECM components and basement membranes (BM), as well as ECM-bound growth factors, MMP-7 and MMP-12 aid in the reconstruction and maintenance of lung tissue. This produces a local release of matrikines (peptides originated from the cleavage of proteins) and signaling molecules that generate a chemotactic gradient that favors local cellular infiltration, activation and differentiation of inflammatory and mesenchymal cells [32]. MMP-7 cleaves DCN [133], a proteoglycan implicated in collagen fibrillization, and inhibits TGF-β1 [33,134,135]. This effect of MMP-7 on TGF-β1 may help to maintain a pro-fibrotic status.

According to these findings, MMP-7 has a dual role as a pro- and antifibrotic mediator, due to its various biological functions related to inflammation, innate immunity, apoptosis and fibroproliferation [34]. MMP-7 enhances neutrophil influx to damage AECs, which promotes the development of fibrosis, and then reverses the fibrotic situation by attracting immunosuppressive leukocytes. Many studies have identified MMP-7 as a potential diagnostic and prognostic biomarker for IPF [136,137,138]. Multiple bioinformatic methods have demonstrated that *mmp7* gene is significantly correlated with the prognosis and occurrence of IPF. [23]. These results are in good agreement with the recent PROFILE (Prospective Observation of Fibrosis in the Lung Clinical Endpoints) study conclusions indicating that serum SP-D and MMP-7 could best differentiate between IPF patients and controls [139,140]. In addition, recent studies using a proximity extension assay identified higher protein levels of MMP-7, IL-6, NOS3 and CASP-8 in PF progressive patients compared to stable patients at follow-up, due to the importance of vascular and remodeling processes linked to progression of the disease [141]. However, recent studies question if MMP-7 is related to PF prognosis [35,142].

#### 3.4.5. MMP-8

MMP-8 (collagenase-2 or neutrophil collagenase) is mainly expressed by polymorphonuclear neutrophils (PMNs). Other cellular types such as activated monocytes, lymphocytes, macrophages, lung epithelial cells, fibrocytes, fibroblasts, natural killer (NK) [143] cells, dendritic cells [144] and mesenchymal cells [36,145,146,147,148] have been described to express MMP-8 at lower levels. PMN activation induces proMMP-8 release, which is stored in PMN-specific granules. TGF-β1 and TNF-α regulate MMP-8 transcriptionally in fibroblasts, and IL-1β and CD-40 ligand in mononuclear phagocytes [148]. Increased levels of MMP-8 have been described in plasma, BAL [125,149,150,151] and lungs of PF patients using multiplexed Luminex immunoassays. *Mmp8*-null mice are protected from bleomycin-induced fibrosis. MMP-8, MMP-9 and TIMP-1 have been selected by multivariable analyses as top candidates to discriminate IPF patients from controls [152].

There are two opposing hypotheses about how MMP-8 supports the PF mechanism. MMP-8, according to one study, can inhibit the synthesis of anti-inflammatory cytokines such as IL-10, promoting inflammation while also increasing collagen formation. As a result, when MMP-8 is absent, MMP-9 and IL-10 levels rise (both of which have anti-fibrotic capabilities) [36]. However, Sun et al. found that IL-10 mediates lung fibrosis development [153]. On the other hand, MMP-8 may also contribute to PF by promoting fibrocyte migration into the lungs (circulating bone marrow-derived mesenchymal progenitor cells displaying CD45 and collagen). These findings were corroborated by in vitro studies. To drive the development of lung fibrosis, IL-10 can induce fibrocyte recruitment via the CCL2/CCR2 (chemokine/chemokine receptor) axis [37]. Although the levels of IFN-inducible protein-10 (CXCL10/IP-10) and MIP1 were raised in *mmp8*-null mice with bleomycin-induced fibrosis, there were no significant variations in the amounts of functional TGF-β1 or IL-10 [146]. However, bleomycin-treated *mmp8^−/−^* mice show more lung inflammation but less lung fibrosis than bleomycin-treated WT mice.

In addition, MMP-8 also exhibits its hypothesized profibrotic activity through the downregulation of MIP1 and IP-10. IP-10 and its receptor CXCR3 suppress fibroblast chemotaxis, which has anti-fibrotic properties [38].

#### 3.4.6. MMP-9

MMP-9 (or gelatinase B) is expressed by neutrophils, AECs, AMs, fibroblasts and fibrocytes. It is overexpressed in BAL and lung tissues of PF patients. ProMMP-9 protein is stored in the tertiary granules of PMNs. In other cell types, transcription factors such as AP-1 and NF-κB regulate MMP-9 expression [154,155]. MMP-9 activity is important in vivo for modifying extracellular matrix components such as collagen IV and laminin in the basement membrane [156]. MMP-9 affects various cellular processes and its expression and secretion are up-regulated in pathological conditions such as cancer and chronic inflammation [39,157,158,159]. MMP-9 has also been demonstrated to activate latent cytokines and growth factors, as well as change myeloid and lymphoid cell trafficking and cell surface protein expression [160]. Concretely, MMP-9 promotes abnormal epithelial repair processes in fibrotic lungs and participates in the development of fibrosis in some experimental situations [39,158,159,160], but its significance in IPF is less clear. Concerning murine fibrosis models, *mmp9*-null mice did not show any change in comparison to WT in bleomycin-induced fibrosis, whereas MMP-9 overexpression in AMs showed a fibrosis reduction [106].

In this sense, results obtained from experimental models have produced some confusing outcomes. In MMP-9 immunohistochemistry, levels are frequently raised in bleomycin-treated mice’s lung tissue homogenates and bronchoalveolar lavage fluids. Surprisingly, the lack of this enzyme did not affect the severity of fibrosis following intratracheal bleomycin [40]. Nevertheless, hypertrophied, and hyperplastic cuboidal epithelial cells, a common epithelial alteration seen in alveolar injury sites, were detected in *mmp9*^+/+^ mice but not in *mmp9*-null mice. The explanation for this discovery is uncertain. However, MMP-9 may help distal airway epithelial cells migrate towards alveolar damaged areas. In addition, *mmp9^−/−^* bleomycin-treated mice are protected from alveolar bronchiolization [40], an aberrant growth of bronchiolar cells in the alveoli seen in experimental PF [161,162,163,164] and severe fibrosis in IPF lungs. By contrast, transgenic overexpression of human MMP-9 in macrophages reduces lung fibrosis [41].

On the other hand, increased circulating MMP-9 could indicate a worse prognosis in IPF, since it has been linked with a higher composite physiologic index [165], and MMP-9 has been described as a potential marker to identify IPF patients in comparison to healthy control [152]. *Mmp9* gene polymorphism has been connected to the effectiveness of immunosuppression in IPF and favors PF and emphysema combination [166]. MMP-9 also encourages airway epithelial cells to produce and activate TGF-β1 [167,168]. In addition, in vitro-related studies demonstrate that MMP-9 is expressed by Thy-1(-) lung fibroblasts with TGF-β1 and enhances fibroblasts migration [36,41,125,169]. As a result, therapies targeting MMP-9 may be able to minimize abnormal lung remodeling, giving a unique clinical strategy for IPF.

A recent study has revealed that MMP-9 modulates airway basal cell (ABC)-like cells in IPF. Blockade of MMP-9 activity with andecaliximab, an anti-MMP-9 antibody, inhibits TGF-β1-induced SMAD2 phosphorylation in IPF patients who have sufficient type 1 IFN expression [170]. Nevertheless, MMP-9’s role in IPF is uncertain nowadays.

#### 3.4.7. MMP-10

MMP-10 (or stromelysin-2) is identified in AECs, macrophages and fibroblasts [42,171,172]. MMP-10 degrades different components of the ECM including proteoglycans, fibronectin and non-fibrillar collagens [173]. MMP-10 has been described in injured and remodeling tissues (skin wound or injured liver and colonic tissue) [173,174,175] since it increases laminin-5 processing in vitro and is required for good adhesion as well as keratinocyte migration [173]. It can activate other MMPs such as proMMP-1, -7, -8 and -9 [176].

MMP-10 and other fibrogenic mediators in the lung such as IL-4, IL-10 and IL-13 are increased in PF in experimental silica models [172,177]. It is demonstrated that TGF-β1 up-regulates MMP-10 in epithelial cells analyzed by RT-PCR. The induction was discovered to be dependent on the myocyte enhancer factor (MEF)-2 transcription factor [178]. In patients with IPF, serum levels of MMP-10 correlate with both clinical deterioration and worse overall survival after six months [42]. Hence, MMP-10 may be a useful IPF biological marker of severity and prognosis.

However, in primary macrophages, RNAi silencing of *mmp10* resulted in a significant reduction in migration. As a result, macrophages implement an aberrant wound healing program associated with pro-inflammatory circumstances [179]. Moreover, MMP-10 induces macrophage conversion from classically activated phenotype M1 to an M2 alternative activation phenotype, which can degrade collagen during skin wound healing (remodeling macrophages). In a skin wound healing model, *mmp10^−/−^* wound deposited higher amounts of collagen than WT [43]. Taking all this into account, the role of MMP-10 could be considered as antifibrotic by increasing macrophage-mediated collagen degradation.

#### 3.4.8. MMP-11

MMP-11 (or stromelysin-3) is activated intracellularly by furin-like protein convertase within the constitutive secretory pathway. MMP-11 has been found in remodeling tissues, such as invasive carcinomas, embryonic development or wound healing [180]. Murine MMP-11’s catalytic domain was discovered to degrade fibrinogen chains [181].

MMP-11 can stimulate Notch signalling [44,45], which in turn can increase *acta2* (α-SMA), *vim* (vimentin) and *col1a1* expression. In contrast, MMP-11 reduces the expression of epithelial marker genes such as *cdh1*. As a result, it promotes FMT and may be linked to the progression of IPF [46]. Nevertheless, the role of MMP-11 in PF and its main biological function(s) remain poorly understood.

#### 3.4.9. MMP-12

MMP-12 (or macrophage metalloelastase) is detected in AM, BECs and smooth muscle cells [182]. MMP-12 is involved in cancer [183] and several chronic pulmonary inflammatory diseases such as chronic obstructive pulmonary disease (COPD), asthma and IPF [184]. MMP-12 can degrade type IV collagen, fibronectin, fibrillin-1, and laminin, among others [185].

Increased MMP-12 levels have been detected in IPF. Since MMP-12 activates the TGF-β1 signaling pathway, this metalloproteinase may contribute to TGF-β1 secretion in IPF [152]. In antibody-mediated Fas-induced lung fibrosis, *mmp12*-null mice have lower expression of profibrotic genes *cry61* (a cysteine-rich ECM protein implicated in fibroblast adherence to ECM) and *egr1* (early growth response factor-1; a zinc-finger transcription factor involved in pulmonary responses to TGF-β1) when compared to WT mice. Hence, MMP-12 is required for the development of the fibrotic phenotype [49]. MMP-12 has a key role in the development of TGF-β1-induced lung fibrosis. TGF-β1 promotes MMP-12, TIMP-1 and inhibits MMP-9 and p21 via Bax- and Bid-dependent pathways. In *mmp12*-null animals, TGF-β1-induced PF is reduced. [47]. The progression of fibrosis has been studied in IL-13/IFN-γ double null mice. This model showed decreased mRNA expression of *mmp12*, *tgfb1* and *timp1* in tissues and reduction in the inflammation and proinflammatory mediators such as TNF-α. MMP-12 decreases collagen deposition in double null mice compared to control [186]. Moreover, during inflammation, MMP-12 modulates inflammatory cytokines such as IL-1β, IL-6, TNF-α, CXCL1 and CXCL3 and macrophage proliferation via the MAPK signaling pathway [48].

Due to type IV collagen degradation activity of MMP-12, the disorder of the basement membrane allows fibroblasts and macrophages to obtain access to the fibrotic tissue and promote PF [187]. Finally, the use of the mTOR inhibitor everolimus in cancer treatment has been linked to the development of PF through the upregulation of MMP-12 expression measured by RT-PCR [188]. However, other authors consider MMP-12 as a repressor of myofibroblast differentiation and lung fibrosis since *mmp12*-null mice showed an enhanced fibrotic response in bleomycin-induced PF compared with WT mice [189].

#### 3.4.10. MMP-13

MMP-13 (or collagenase-3) is mainly identified in BECs and AECs, AM and in interstitial spaces during the inflammation and fibrosis resolution of IPF. MMP-13 is reported to be upregulated in IPF compared to control lungs [50,53,190]. MMP-13 is known for its collagenolytic activities, particularly with respect to fibrillar type I and II collagens. [102,191]. The recombinant catalytic domain of MT1-MMP (MMP-14) efficiently activates procollagenase-3 (proMMP-13) through active gelatinase A (MMP-2) [192]. MMP-13 has recently been reported to have an important role in hepatic fibrosis [191,193]. However, its involvement in lung fibrosis is still unknown.

In both mice and human studies, MMP-13 seems to be implicated in the control of ECM and collagen deposition since it degrades collagen in sites of dense fibrosis. In *mmp13*-null mice, the lack of this MMP may result in ECM degradation problems and postpone fibrosis resolution [50]. However, excessive MMP-13 functions can promote the formation of honeycomb cysts [53]. In a bleomycin-mediated PF model, *mmp13*-null animals suffered increased inflammation and severity with a high neutrophilic response compared to WT mice [50,52,53]. It was produced by an increase in macrophage infiltration and significant alterations in proinflammatory cytokines. In addition, *mmp13*-null animals had more severe and persistent PF compared to WT mice. The expression of α-SMA is increased in *mmp13*-null mice compared to WT animals. All these facts suggest that MMP-13 is an antifibrotic protease [50]. Nevertheless, studies in a radiation-induced lung fibrosis model revealed that *mmp13*-null mice exhibit reduced acute pulmonary inflammation as is evidenced by reduced alveolar septi and lung architectural remodeling in histology. Which is to say, *mmp13*-null mice in IPF reveal contradictory phenotypes of inflammatory and fibrotic responses [50,51].

In conclusion, MMP-13’s impact on the inflammatory response is crucial to the severity of IPF development. However, the role of MMP-13 in IPF is uncertain, and the findings are contradictory.

#### 3.4.11. MMP-14

MMP-14 (or membrane-type 1 matrix metalloproteinase (MT1-MMP)) belongs to the subfamily of the membrane-type MMP family. It was discovered as a proMMP2 activator, expressed on the surface of invasive cancer cells [108]. MMP-14 is overexpressed in AECs of IPF lungs, mainly in hyperplastic cuboidal type 2 pneumocytes and bleomycin-induced lung fibrosis [56,194,195]. It processes types I–III collagen [196]. MT1-MMP is required for pulmonary fibroblast migration in three-dimensional (3-D) cross-linked type I collagen hydrogels [197].

MT1-MMP may increase fibrotic responses by activating TGF-β1 and/or limiting normal repair processes in the wounded lung [198]. In a fibrotic lung bleomycin-induced mice model, the MMP-14/Nogo-B (a member of the endoplasmic reticulum protein family) pathway acts as a novel driving factor of the EMT process, which can enhance the EMT of cells by liberating TGF-β1 [54].

By contrast, in vitro and in vivo experiments deleting or inhibiting MT1-MMP expression in fibroblasts or tumor cells leads to a loss of collagenolytic and invasive activity. As a result, MT1-MMP is the main cell-associated proteinase required for normal or cancerous cells to invade and ECM remodeling [199,200]. Therefore, a lack of this MMP increase the severity of fibrosis by decreasing collagen destruction compared to WT mice [59]. Similar results have been reported in stromal fibroblasts of *mmp14*-null mice [201]. In this case, deletion of *mmp14* in adult mice fibroblast induces fibrotic skin phenotype since they are not able to process collagen type I. Moreover, bleomycin-induced experimental lung fibrosis is exacerbated by epithelial MMP-14 deficiency since it reveals abnormal proteolytic processing of ECM, a process associated with an epithelial senescence phenotype [55] related to the aggravation of fibrosis [202]. These epithelial senescence cells overexpress TGF-β1 and increase profibrotic markers in fibroblasts such as *acta2* and *fn1* (Fibronectin) by RT-PCR [56].

#### 3.4.12. MMP-19

The catalytic domain of MMP-19 has a distinctive structure (it lacks Asp, Tyr and Gly residues located close to the zinc-binding site in collagenases, the fibronectin-like domain of gelatinases, the transmembrane domain of MT-MMPs and the furin-activation sequence) (Table 1), chromosomal location and tissue distribution [203,204]. MMP-19 is produced highly in fibroblasts, endothelial cells, monocytes and macrophages [205]. Specifically, this enzyme is differentially expressed in AECs adjacent to fibrotic regions in IPF lungs [58,59].

Some studies have analyzed its role in vivo using the bleomycin-induced fibrosis mice model and the fibrotic response has been increased in animals lacking this MMP [58]. Moreover, *mmp19^−/−^* lung fibroblast develops an exacerbated profibrotic gene expression (α11 integrin, *itga11*, a receptor of collagen, fibrillar, among others) compared to control mice [59,206]. MMP-19 has a strong regulatory effect on several profibrotic processes in lung fibroblasts. Molecular abrogation of this enzyme results in a dysregulated ECM synthesis, growth rate, transmigration capacity, cell–matrix interaction, as well as increased type I collagen production in wound-healing response [59]. The study of FMT by α-SMA expression reveals that *mmp19*-null mice express a higher amount of this marker compared with WT mice. Hence, this MMP plays an important role in tissue remodeling and fibrogenesis [59]. Furthermore, MMP-19 mediates its antifibrotic activities by increasing the expression of cyclooxygenase 2 (COX2), an enzyme involved in the synthesis of prostaglandin E2. This prostaglandin suppresses fibroblast proliferation, migration and collagen synthesis [59,207,208,209]. *mmp19*-null fibroblasts significantly increase proliferative and migratory activities. Reintroducing MMP-19 recombinant protein decreased gene expression levels of *col1α1*, *mmp14*, and *itga11*, among others, reaching similar levels to WT fibroblasts. *Itga11* is induced by TGF-β1 contributing to the differentiation of fibroblasts to myofibroblasts. Moreover, *mmp19*-null mice upregulate gene expression of *lox11*, a lysyl oxidase overexpressed in fibrotic development since it plays a role in the formation of crosslinks in collagens and elastin [57,59]. This increment confirms the profibrotic character of *mmp19*-deficient fibroblasts.

MMP-19 also orchestrates immune responses. In vivo studies with *mmp19*-null mice propose MMP-19 as an important factor in cutaneous immune responses and influences the development of T cells since *mmp19*-null mice had an impaired T cell-mediated immune response [210]. 

In conclusion, it seems clear that MMP-19 is a potent antifibrotic protease.

#### 3.4.13. MMP-28

MMP-28 (or epilysin) is the last member of the MMPs. Its structure includes a prototype domain of MMP and is activated intracellularly by a furin-like proprotein convertase [60]. Epilysin is expressed constitutively in numerous tissues especially in AECs and BEC [211] and *mmp28* gene expression is elevated in IPF patients’ lungs [61]. MMP-28 has been identified not only in the cytoplasm but also in the AEC nucleus. By contrast, in BECs, MMP-28 staining is mostly apical [211]. Moreover, MMP-28 is also expressed by lung macrophages during the development and maintenance of fibrosis. This MMP also takes part in the development and regeneration of the nervous system [212], homeostasis in human epithelial [213] and its importance in several pathological states has been reported such as gastric carcinoma [214,215].

MMP-28’s relevance in IPF is still unknown. However, some studies have revealed increased levels of MMP-28 in serum from patients with IPF compared with non-IPF patients measured by ELISA [63]. Additionally, in vitro studies in human and rat AEC found that this enzyme enhances lung epithelial growth rate, migration, proliferation, and provides protection from apoptosis among others [61]. The roles of MMP-28 in migration and proliferation depend on its catalytic activity, as E to A mutations yielding a catalytically dead form of the protease do not exhibit these behaviors [60,61,212]. Overexpression of MMP-28 results in upregulation of MT1-MMP, MMP-9 by Western blotting, and encourages the collagen invasive activity of A549 cells [212,215,216]. It has been noticed that MMP-28 upregulation protected AEC from apoptosis using bleomycin as an apoptotic stimulus, whereas silencing the enzyme decreased proliferation rate and delayed wound closing by scratch wound-healing assay and transmigration over type I collagen [211]. Nevertheless, the pathologic consequences of these in vivo actions remain to be established. In addition, there is some evidence that MMP-28 promotes a TGF-β1-dependent mechanism that contributes to EMT in cancer cells, a process related to IPF [63,217].

Other in vivo experiments have suggested that epilysin has a role in COPD. *mmp28^−/−^* mice are protected from inflammation and emphysema caused by tobacco smoke since MMP-28 is critical for leukocyte recruitment into chronic smoke-exposed lungs; indeed, neutrophil adhesion receptors and chemokines are reduced in *mmp28^−/−^* mice [216]. Furthermore, MMP-28 induces macrophages to convert from classically activated phenotype M1 to an M2 alternative activation phenotype, which increases IPF in bleomycin-treated animals [62] and enhances fibroblast proliferation and collagen production [218]. Finally, it has been proposed that MMP-28 could be considered as a putative diagnostic biomarker in IPF [63]. However, more evidence is needed to clarify the clinical utility of MMP-28.

#### 3.4.14. Other MMPs with Possible Involvement in Pulmonary Fibrosis

Other MMPs have been shown to play a role in the remodeling process associated with tumor pathogenesis. However, even less is known about the roles of these MMPs in IPF.

MMP-15, MMP-16, MMP-17, MMP-24 and MMP-25 are membrane-type MMP. They have been linked to cell invasion and angiogenesis [219,220,221,222]. MMP-16 and MMP-24 can transform proMMP-2 to active MMP-2, and MMP-16 can degrade numerous ECM components [223,224]. Downregulation of MMP-16 inhibits EMT by increasing the expression of *cdh1* while repressing mesenchymal markers *vim* and *cdh2* [225]. MMP-17 could be involved in the activation of membrane-bound precursors of growth factors or inflammatory mediators such as TNF-α [11]. MMP-21 is overexpressed in cancer cells compared to normal epithelial tissue and is involved in cell adhesion, migration and invasion [226]. MMP-26, or matrilysin-2, has an essential role in the local invasion through coordination with MMP-9 [227,228].

All these reports support the general idea that other MMPs or proteases may modulate PF, since they participate in processes that accompany, activate or perpetuate the fibrotic response and the remodeling turnover in other pathologies such as cancer. Therefore, further investigation into the role of these MMPs in fibrosis is needed to provide a better insight into their biological and clinical value.

#### 3.4.15. TIMPs in Pulmonary Fibrosis

Deletion of TIMPs in mice has been studied to explore their role in normal and pathological processes. *timp1^−/−^*, *timp2^−/−^* and *timp4^−/−^* mice display apparently normal phenotypes but exhibit interesting features associated with the absence of TIMPs, such as impaired learning due to abnormal neural plasticity in *timp1^−/−^* mice [229], no proMMP2 activation in *timp2^−/−^* mice [230], and increased mortality after myocardial infarction induction in *timp4^−/−^* mice [231]. It is not completely clear if these mice alter their MMP and/or TIMP expression and activity to compensate for the lack of a specific TIMP. However, *timp3^−/−^* mice exhibit spontaneous air space enlargement in the lungs, but there is no increase in inflammatory cell infiltration or evidence of fibrosis in comparison with controls [232]. This TIMP is described to reside within the ECM to inhibit its degradation by MMPs or ADAMs.

In bleomycin-induced PF, *timp1^−/−^* mice showed no differences in fibrosis induction but increased inflammation after lung injury [233]. *Timp3^−/−^* mice showed a more severe fibrotic response resulting from persistent inflammation due to increased neutrophil influx [234]. Therefore, TIMP-3 seems to be antifibrotic in mice. The imbalance between TIMP-1/-2 and MMP-2/-9 has been proposed to regulate ECM after bleomycin treatment but the results are sometimes contradictory [235,236,237,238,239].

After TGF-β1 stimulation, TIMP-3 is strongly stimulated in fibroblasts by p38 activation. In addition, this TIMP is found in fibroblast foci in IPF [194]. However, it is not clear if human IPF primary fibroblasts upregulate TIMPs compared to controls [194,240,241].

Other histological studies of IPF patients show the presence of TIMP-1 in interstitial macrophages, TIMP-2 in fibroblast foci, TIMP-3 in the elastic lamina in vessels and TIMP-4 in epithelial and plasma cells, supporting the idea that reduced collagen degradation is fundamental to this disorder [93]. However, MMPs and TIMPs are frequently observed in the same locations, so the role of MMPs and TIMPs in vivo is complex and still needs further research [242,243].

A multicenter study has shown that circulating MMPs and TIMPs were broadly elevated among patients with IPF, mainly TIMP-1, but not TIMP-2. In this study, TIMP-3 was not evaluated [152].

All the evidence points to TIMPs playing an important role in PF but not only by counteracting MMPs activity. Their participation in IPF and the inflammatory response of the lung should be clarified in the future.

## 4. EMMPRIN/CD147 and Its Role in Pulmonary Fibrosis

### 4.1. Introduction

Cluster of differentiation 147 (CD147), also known as EMMPRIN (extracellular matrix metalloproteinase inducer), is a transmembrane glycoprotein that belongs to the immunoglobulin superfamily (IgSF) [244]. It was first isolated in the Biswas lab from the LX-1 human pulmonary carcinoma cell line and named tumor cell-derived collagenase stimulatory factor (TCSF) because it increased the production of matrix metalloproteinases in nearby normal fibroblasts, allowing malignant cells to migrate more easily through the extracellular matrix, implying a role in tumor progression [245]. Then, it was renamed EMMPRIN based on its functional participation in MMP induction [246]. It is also known as basigin, encoded by the *BSG* gene located on human chromosome 19 (p13.3) [247].

Other alternative names are leukocyte activation antigen M6, blood group antigen OX47 in rat, 5A11 antigen in chicken, neurothelin, HT7 and hepatoma-associated antigen (HAb18G) in human [248,249].

EMMPRIN/CD147 can stimulate the synthesis of MMP-1 (collagenase), MMP-2 (gelatinase A), MMP-3 (stromelysin-1), MMP-9 (gelatinase B) and MMP-14 by mesenchymal cells [250,251]. Consequently, it has been implicated in the development of cancer, tissue remodeling and fibrosis with a variety of inflammatory diseases in multiple investigations [252,253]. CD147 and its partners have been used as diagnostic and therapeutic markers in cancer and inflammatory diseases [252]. In addition, studies in vitro have demonstrated that the effect of CD147 is not limited to tumor-endothelial cell and tumor-fibroblast heterophilic interactions. CD147 can directly stimulate MMP production in both tumor cells and fibroblasts individually [254].

### 4.2. Structure and Molecular Interactions of EMMPRIN/CD147

**Figure 1:**
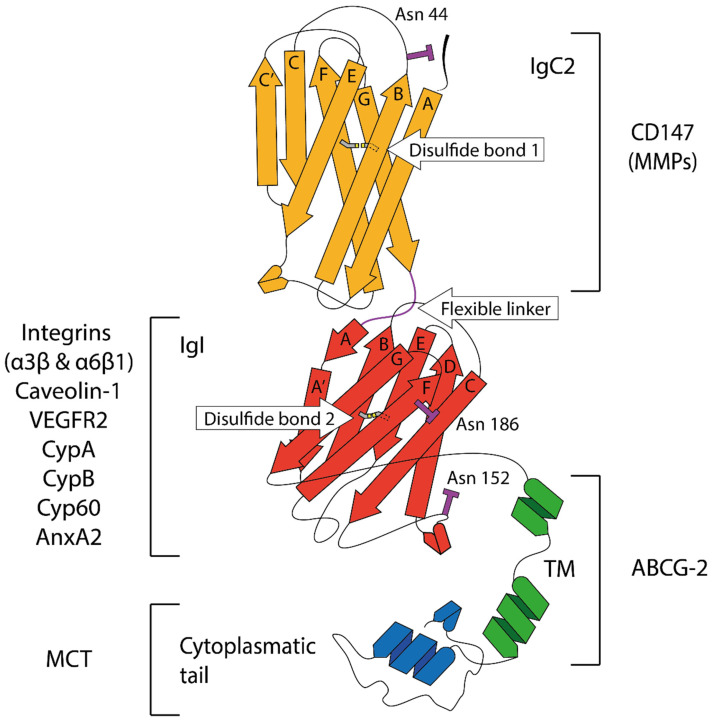
Structure of CD147/bsg-2 with the two Ig domains (IgC2 and IgI) connected by a flexible linker, the transmembrane domain and the cytoplasmatic domain. The cartoon shows the diagrammatic representation of the molecular structure of CD147 and molecules potentially interacting with each domain based on previous reports [249,250,255]. Letters label molecular strands forming Ig domains. The N-terminal domain (IgC2, orange) has a disulfide bond connecting strand B and F (between C41 and C87, respectively) and an N-linked glycosylation site at Asn-44 at the end of strand B. This domain is responsible for homophilic interactions and influences MMP activity. The C-terminal domain (IgI, red) has a disulfide bond connecting B and F strand (between C126 and C185, respectively) and two potential glycosylation sites at Asn-152 and Asn-186. The flexible linker is shown in purple, the transmembrane domain (TM) in green and the cytoplasmatic domain in blue.

CD147 or basigin comprises four different isoforms by alternative promoters and splicing, named CD147/bsg-1, -2, -3 and -4 [253]. CD147/bsg-1 is a retina-specific isoform with three Ig-like domains. CD147/bsg-3 and -4 contain a single Ig-like domain. The most abundant and best-characterized isoform is CD147/bsg-2, which comprises two Ig-like domains (Figure 1). This review will be focused on CD147/bsg-2 (referred as CD147) unless specified otherwise.

CD147 is a single chain type I transmembrane protein with homology to both the Ig variable domain (V) and MHC-II β-chain [246,248] and contains an N-terminal IgC2 domain (Domain 1 or Ig1) and a C-terminal IgI domain (domain 2 or Ig2), which are connected by a 5-residue flexible linker containing a GPP motif (Figure 1) [256]. This domain organization is unique in the IgSF family and provides great mobility to make homo and heterophilic interactions for its multi-functional role [256]. The TM domain contains highly conserved glutamic acid and leucine zipper-like sequences within the hydrophobic sequence of the transmembrane domain [255]. These sequences contribute to the anchoring of CD147 to the cell membrane. The cytoplasmic domain, TM domain, 5-residue flexible linker, cysteine residues, and asparagine glycosylation sites are well-conserved sequences across species [248]. CD147 exhibits three potential glycosylation sites: one for Ig1 at N44 at the end of strand B, and two for Ig2 at N152 at the middle of the C’D loop and N186 at strand F (Figure 1) [249]. The degree of glycosylation modifies its function and its proper folding [257,258]. Thus, CD147 has been classified into highly glycosylated CD147 (HG-CD147, molecular weight 45–65 kDa) and low glycosylated CD147 (LG-CD147, molecular weight 32 kDa). The induction of MMP-1 and MMP-2 has been associated with HG-CD147 [259], which promotes tumor invasion and migration. LG-CD147 associates with caveolin-1 to inhibit CD147 self-aggregation and its conversion from LG-CD147 to HG-CD147. Consequently, caveolin-1 blocks MMP induction mediated by HG-CD147 [259]. Of note, caveolin-1 also interacts with MMP-2, and this interaction seems to inhibit MMP-2 activity [260]. However, the contribution of the CD147 glycosylation to MMPs induction is controversial since other authors have found MMP induction, even in LG-CD147 [261,262]. Probably, both LG- and HG-CD147 contribute to MMP activity but principally HG-CD147 [248].

In addition to glycosylation, CD147 function is mediated by its homophilic and heterophilic molecular interactions. Intercellular homophilic interactions are produced on opposing cells or on nearby cells after membrane vesicle shedding. CD147 acts as its own receptor due to homophilic interactions in a *trans* manner to stimulate MMP production and this interaction is mediated by the N-terminal Ig domain [263], dependent on the CD147 glycosylation state [264]. In addition, homophilic *cis* interactions are produced at the intracellular plasma membrane (*cis*-recognition) to form homo-oligomers [255].

CD147 interacts with a wide range of proteins by heterophilic molecular interactions to carry out several functions in physiological and pathological conditions. Mostly, they have been studied in the context of cancer. Monocarboxylate transporters (MCTs, such as MCT1 and MCT4) are responsible for transmembrane transport of lactate, playing a key role in cancer cells to avoid the typical intracellular accumulation of protons related to tumor metabolism. Interestingly, they are tightly associated with CD147 for pH regulation and cancer progression. In this context, MCT-CD147 interaction may have clinical significance [265]. Moreover, CD147 stimulates hyaluronan synthesis and interaction of hyaluronan with its receptors, in particular CD44 and LYVE-1, which in turn result in the activation of multiprotein complexes containing members of the membrane-type matrix metalloproteinase, receptor tyrosine kinase, ABC drug transporter or MCT families within lipid raft domains [266]. Again, these interactions promote cancer progression and cancer stem cell malignancy.

Interactions with proteins of the cyclophilin family have been reported to play a role in protein trafficking and inflammatory diseases such as acute or chronic lung diseases. CD147 acts as a signaling receptor for both cyclophilin A (CypA) and B (CypB), strong chemotactic agents [267]. In cancer, CypA-CD147 interaction induces invasion of tumor cells [268] and stimulates cell proliferation [269]. Similarly, cyclophilin 60 (Cyp60) interacts with CD147 for its translocation to the cell surface [270]. Cyp60 and MCT seem to help CD147 to be expressed on the plasma membrane.

Other proteins forming complexes with CD147 are integrins. For example, integrin α3β1 and α6β1 interact with CD147, contributing to tumor invasion by the FAK signaling pathway activation [271]. These interactions modulate CD147 function and promote CD147 clustering.

CD147 is implicated in angiogenesis by regulating VEGF expression. Of note, CD147 acts as a co-receptor of VEGFR2 in both endothelial and tumor cells. Therefore, VEGFR-2-CD147 complexes enhance VEGF functions related to angiogenesis and tumor progression [272].

In addition to the mentioned above caveolin-1, emerging evidence suggests that other proteins interact with CD147 modulating their function and MMP induction such as annexin A2 (ANXA2) [273] galectin-3 (GAL3) [274] tumor necrosis factor receptor-associated factor (TRAF6) [275], the transporter ABCG2 [276] and MT1-MMP [262] (Figure 1). Certainly, more ligands will be discovered in the future to interact with CD147, helping us to understand its complex function in physiological and pathological conditions.

### 4.3. Expression of CD147

CD147 levels have been found to be elevated in a variety of malignant tumors and have been linked to tumor progression in both experimental and clinical settings. CD147 is overexpressed in more than 60% of human lung cancer and in most lung carcinomas [277,278,279]. In addition, expression has been found in various malignancies such as laryngeal carcinomas [280], breast carcinomas [281], lymphoma [282], hepatoma [283], and malignant pigment cell lesions [284]. Recent studies associate CD147 expression with metastatic progression in osteosarcoma [285] and describe CD147 as a promising marker for the detection of circulating tumor cells in Small Cell Lung Cancer [286]. Furthermore, the discovery of CD147 in non-tumoral tissues suggests that it may have a role in other physiological and pathological circumstances producing increased MMP expression. CD147 levels were shown to be higher in smokers’ BAL [287] and have been detected in non-neoplastic processes such as human atheroma [288], ventilator-induced lung injury in rats [289], left ventricular myocardium failure [290] and normal and ulcerated corneas [254]. CD147 is expressed in a range of embryonic tissues including the lung [291], but only at low levels in normal adult lungs and other tissues [279,292]. Expression of CD147 in the trophectoderm, embryo proper and uterine endometrium seems key in the intercellular recognition during implantation, since the majority of *Bsg^−/−^* mice died around the time of implantation. Interestingly, half of the survivors died before 1 month after birth due to interstitial pneumonia, and the other half were sterile and small [293].

### 4.4. Role of CD147 in Pulmonary Fibrosis

There are, at least, four reasons to support the participation of CD147 in PF: (1) Fibroblasts were firstly described as effectors of CD147 activity in the earliest Biswas’s experiments [246]; (2) as mentioned above, CD147 induces secretion of MMPs, which play a critical role in PF; (3) direct interactions of CD147 with other proteins observed in cancer are responsible for functions that also modulate fibrosis development; and (4) PF shares similarities with lung cancer progression and the presence of stroma and fibroblasts in lung cancer worsens clinical outcome [294]. However, studies of CD147 in PF are scarce and the role of CD147 in the disease remains partially unknown. The importance of CD147 in fibrosis is also observed in other organs such as the liver [295] and stroma in cancer [296].

The presence of CD147 in PF has been studied by specific immunoreactivity on lung fibrotic tissue obtained from IPF patients and in the bleomycin-induced model of PF in mice. In both scenarios, CD147 is overexpressed in areas of active fibrosis. CD147 was mostly seen in pneumocytes and tissue macrophages mainly in their plasma membranes. Consequently, BAL showed CD147 expression in humans and mice. However other cell types such as fibroblasts and myofibroblasts, endothelial cells, vascular smooth muscle cells and airway smooth muscle cells did not show CD147 staining [297,298]. The evidence suggests that fibroblasts themselves are not a clear source of CD147 but may respond to it by modulating their MMP expression, as has been described in the context of cancer. However, the specific relevance of CD147 in MMP expression associated with PF is not well-studied. Of note, TIMPs expression is not modified by CD147 [254]. It seems reasonable that CD147 modifies the protease/antiprotease balance in favor of proteases, but the specific impact on it remains undefined in PF.

CD147 induces FMT in fibroblasts from lung [299], skin [300] or breast cancer [296], a process that seems to be MMPs-independent. In these studies, fibroblast CD147 overexpression or fibroblast treatment with recombinant CD147 is correlated with higher levels of α-SMA and other myofibroblast features such as increased collagen gel contraction or resistance to apoptosis compared to control. In addition, TGF-β1 favors the expression of CD147 by fibroblasts in a dose-dependent manner by the activation of the canonical Wnt/β-catenin-signaling pathway [299]. CD147 may interact with several molecules to modulate the fibrosis process. For example, recent study suggests that CD44s/CD147 colocalization and interaction are crucial for regulating the mechanical strain required for α-SMA incorporation into F-actin stress fibers, which modulates FMT, driven by TGF-β1 [301]. The specific role of CD147 expression in PF in fibroblast may be elucidated by studying models of fibrosis in a recently described fibroblast-specific CD147 null mouse model, which showed no obvious differences in the morphology of the lung or other organs compared to controls [302].

Regarding macrophages, CD147 promoted M1 macrophages, which in turn induced the differentiation of Th17 cells in bleomycin-induced lung fibrosis [303]. In this model, the inhibition of CD147 by antibodies reduced PF in mice. Additional reports support the profibrotic role of CD147 in the bleomycin-induced model. Thus, CD147 plays a key role in epithelial–mesenchymal interactions during diffuse alveolar injury and repair, resulting in changes in tissue architecture [298,304]. Furthermore, these findings rule out any classical functional connection between CD147 and caveolin-1 in the AECs [304]. Moreover, it has been demonstrated that inhibition of CD147 can reduce collagen-I synthesis in lung fibroblasts incubated with cultured pleural mesothelial cells (PMCs) and prevent bleomycin-induced PF in an MMPs-dependent process [305]. Consequently, CD147 causes ECM degradation via regulating MMP synthesis, or it causes ECM deposition by inducing myofibroblast differentiation. CD147 and the nature of cell–cell interactions may influence the transition from ECM fibrosis to lysis [252].

All of these results evidence that CD147 could be directly related to fibrosis processes and CD147 is closely linked to the accumulation and remodeling of the extracellular matrix producing PF [297]. Overexpression of CD147 in lung fibroblasts causes an antiapoptotic and profibrotic phenotype and the induction of FMT, which may contribute to the prolonged fibro-proliferative state seen in IPF [299].

## 5. Concluding Remarks and Future Perspectives

**Figure 2:**
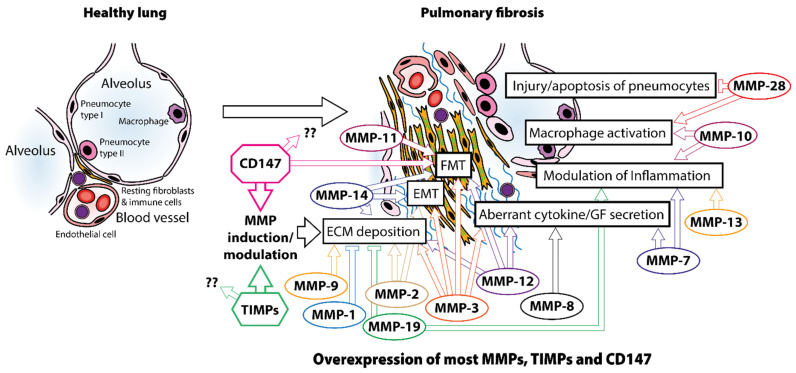
Summary illustration where most important MMPs, TIMPs and CD147 are shown in PF. The arrows point to their main role in most representative features of fibrosis, discussed in this manuscript. Fibroblast foci are presented in PF (right) with fibroblast (orange), myofibroblasts (orange plus green alpha-SMA fibers inside) and collagen deposition (blue wavy lines). Other cells accompany and modulate fibrosis, such as macrophages (wavy cell in purple), immune cells (purple), endothelial cells (pale red) and pneumocytes (pink). EMT = epithelial-to-mesenchymal transition. FMT = fibroblast-to-myofibroblast transition. GF = growth factors. ECM = extracellular matrix.

PF is a complex condition in which several cells interact leading to aberrant ECM deposition and fibroblast/myofibroblast proliferation. Fibrosis development has an important impact on the biomechanical traits of the lungs. TGF-β1 and other profibrotic factors perpetuate this situation promoting a chronic fibrotic scenario. Paradoxically, proteases and antiproteases such as MMPs and TIMPs are generally induced in the disease to create a disrupted protease balance in which most well-studied MMPs promote fibrosis (Figure 2). Unfortunately, there are still several MMPs with an unknown or unclear role in the disease. A given MMP may be pro-fibrotic in one context and anti-fibrotic in another, and this contextual sensitivity includes not only stages of disease progression, but also spatial tissue context, genetic background, inflammatory status, and other facets of tissue microarchitecture that are rarely considered such as mechanical stress and redox state. MMP participation in the development of fibrosis will be clarified in the future. Of note, the use of a single MMP or TIMP as a therapeutic target may have beneficial and detrimental effects depending on its role and the phase of the disease. The lack of highly specific inhibitors/modulators for a particular MMP or TIMP that could reach effective concentrations in the fibrotic tissue hinders this objective. In addition, most of the advances obtained in our understanding of MMPs come from in vitro studies, which have obvious limitations and explain, at least in part, some of the apparently paradoxical results discussed. The complexity of extracellular protein–protein interaction networks and extensive post-translational regulatory mechanisms modulating MMP activity in vivo demands more and better in vivo experimental analysis. Given the extensive redundancy between MMPs and TIMPs in mice (illustrated by the phenotypic normalcy of single and even double MMP knockouts), the induction of fibrosis in knock-out mouse models has limitations for elucidating the underlying biological functions of these proteases.

In the context of cancer, CD147 was discovered as an inducer of MMPs in fibroblasts, promoting tumor progression and invasion. Over the last years, several molecules have been described to interact with CD147 to provide additional functions in cancer that potentially may have an impact on PF. CD147 emerges as a profibrotic factor not only by stimulating MMPs but also by inducing FMT (Figure 2). As in the history of cancer research, other functions will be associated with its expression in the future. The lack of clinical efficacy points out the need for new therapies to enhance or replace actual treatment in IPF. The evidence summarized in this review indicates that CD147 is a promising druggable target in PF. The strategy for inhibiting its function would go through the targeting of protein–protein-interacting interfaces by peptide or monoclonal antibodies over small molecules due to its larger binding interface [249]. The authors encourage the scientific community to study the role of CD147 in the fibrotic process, and its potential use as a prognostic biomarker and/or therapeutic target in IPF.

## Figures and Tables

**Table 1:** Classification of matrix metalloproteinases and their role in pulmonary fibrosis.

MMP	Substrate Classification [11]	Structure (N-Terminal Left, C-Terminal Right)	Role in PF	References
MMP-1	Collagenase-1	SP-ProP(SH)-CatZn^2+^-H-PEX	Unclear	[21,22,23,24]
MMP-2	Gelatinase A	SP-ProP(SH)-Cat(Fn-Fn-Fn)Zn^2+^-H-PEX	Profibrotic	[25,26,27,28]
MMP-3	Stromelysin-1	SP-ProP(SH)-CatZn^2+^-H-PEX	Profibrotic	[29,30,31]
MMP-7	Matrilysin-1	SP-ProP(SH)-CatZn^2+^	Profibrotic and Antifibrotic	[32,33,34,35]
MMP-8	Collagenase-2	SP-ProP(SH)-CatZn^2^^+^-H-PEX	Profibrotic	[36,37,38]
MMP-9	Gelatinase B	SP-ProP(SH)-Cat(Fn-Fn-Fn)Zn^2^^+^-H-PEX	Unclear	[39,40,41]
MMP-10	Stromelysin-2	SP-ProP(SH)-CatZn^2+^-H-PEX	Unclear	[42,43]
MMP-11	Stromelysin-3	SP-ProP(SH)Fu-CatZn^2+^-H-PEX	Profibrotic	[44,45,46]
MMP-12	Macrophage metalloelastase (Others)	SP-ProP(SH)-CatZn^2+^-H-PEX	Profibrotic	[47,48,49]
MMP-13	Collagenase-3	SP-ProP(SH)-CatZn^2+^-H-PEX	Unclear	[50,51,52,53]
MMP-14	MT1-MMP	SP-ProP(SH)Fu-CatZn^2+^-H-PEX -TM-Cy	Unclear	[54,55,56]
MMP-15	MT2-MMP	SP-ProP(SH)Fu-CatZn^2+^-H-PEX -TM-Cy	Unknown	
MMP-16	MT3-MMP	SP-ProP(SH)Fu-CatZn^2+^-H-PEX -TM-Cy	Unknown	
MMP-17	MT4-MMP	SP-ProP(SH)Fu-CatZn^2+^-H-PEX -GPI	Unknown	
MMP-19	Others	SP-ProP(SH)-CatZn^2+^-H-PEX	Antifibrotic	[57,58,59]
MMP-20	Others	SP-ProP(SH)-CatZn^2+^-H-PEX	Unknown	
MMP-21	Others	SP-ProP(SH)VnFu-CatZn^2+^-H-PEX	Unknown	
MMP-23	Others	N-II-ProP(SH)Fu-Cat-CA-IgG-Like	Unknown	
MMP-24	MT5-MMP	SP-ProP(SH)Fu-CatZn^2+^-H-PEX-TM-Cy	Unknown	
MMP-25	MT6-MMP	SP-ProP(SH)Fu-CatZn^2+^-H-PEX-GPI	Unknown	
MMP-26	Matrilysin 2	SP-ProP(SH)-CatZn^2+^	Unknown	
MMP-27	Others	SP-ProP(SH)-CatZn^2+^-H-PEX	Unknown	
MMP-28	Others	SP-ProP(SH)Fu-CatZn^2+^-H-PEX	Profibrotic	[60,61,62,63]

SP = signal peptide, ProP(SH) = propeptide domain with cysteine, Fu = furin-like proprotein convertase recognition sequence, CatZn^2+^ = catalytic metalloproteinase domain containing Zinc^2+^, H = Hinge region, PEX = Hemopexin domain, TM = transmembrane domain, GPI = glycophosphatidyl domain, Cy = Cytoplasmic tail, Fn = Fibronectin type II domain, Vn = Vitronectin-like insert, CA = cysteine array, IgG = immunoglobulin-like domain, N-II = type II transmembrane domain (N-terminal signal anchor).

## Data Availability

Not applicable.

## References

[1] López-Muñiz Ballesteros B., López-Herranz M., Lopez-De-andrés A., Hernandez-Barrera V., Jiménez-García R., Carabantes-Alarcon D., Jiménez-Trujillo I., de Miguel-Diez J. (2021). Sex Differences in the Incidence and Outcomes of Patients Hospitalized by Idiopathic Pulmonary Fibrosis (IPF) in Spain from 2016 to 2019. J. Clin. Med..

[2] Bonnans C., Chou J., Werb Z. (2014). Remodelling the Extracellular Matrix in Development and Disease. Nat. Rev. Mol. Cell Biol..

[3] Burgstaller G., Oehrle B., Gerckens M., White E.S., Schiller H.B., Eickelberg O. (2017). The Instructive Extracellular Matrix of the Lung: Basic Composition and Alterations in Chronic Lung Disease. Eur. Respir. J..

[4] Liu F., Mih J.D., Shea B.S., Kho A.T., Sharif A.S., Tager A.M., Tschumperlin D.J. (2010). Feedback Amplification of Fibrosis through Matrix Stiffening and COX-2 Suppression. J. Cell Biol..

[5] Hinz B. (2012). Mechanical Aspects of Lung Fibrosis: A Spotlight on the Myofibroblast. Proc. Am. Thorac. Soc..

[6] Gabasa M., Duch P., Jorba I., Giménez A., Lugo R., Pavelescu I., Rodríguez-Pascual F., Molina-Molina M., Xaubet A., Pereda J. (2017). Epithelial Contribution to the Profibrotic Stiff Microenvironment and Myofibroblast Population in Lung Fibrosis. Mol. Biol. Cell.

[7] Puig M., Lugo R., Gabasa M., Giménez A., Velásquez A., Galgoczy R., Ramírez J., Gómez-Caro A., Busnadiego Ó., Rodríguez-Pascual F. (2015). Matrix Stiffening and Β1 Integrin Drive Subtype-Specific Fibroblast Accumulation in Lung Cancer. Mol. Cancer Res..

[8] Lagares D., Santos A., Grasberger P.E., Liu F., Probst C.K., Rahimi R.A., Sakai N., Kuehl T., Ryan J., Bhola P. (2017). Targeted Apoptosis of Myofibroblasts with the BH3 Mimetic ABT-263 Reverses Established Fibrosis. Sci. Transl. Med..

[9] Giménez A., Duch P., Puig M., Gabasa M., Xaubet A., Alcaraz J. (2017). Dysregulated Collagen Homeostasis by Matrix Stiffening and TGF-Β1 in Fibroblasts from Idiopathic Pulmonary Fibrosis Patients: Role of FAK/Akt. Int. J. Mol. Sci..

[10] Galgoczy R., Pastor I., Colom A., Giménez A., Mas F., Alcaraz J. (2014). A Spectrophotometer-Based Diffusivity Assay Reveals That Diffusion Hindrance of Small Molecules in Extracellular Matrix Gels Used in 3D Cultures Is Dominated by Viscous Effects. Colloids Surf. B. Biointerfaces.

[11] Cui N., Hu M., Khalil R.A. (2017). Biochemical and Biological Attributes of Matrix Metalloproteinases. Prog. Mol. Biol. Transl. Sci..

[12] Gross J., Lapiere C.M. (1962). Collagenolytic Activity in Amphibian Tissues: A Tissue Culture Assay. Proc. Natl. Acad. Sci. USA.

[13] Puente X.S., Sánchez L.M., Overall C.M., López-Otín C. (2003). Human and Mouse Proteases: A Comparative Genomic Approach. Nat. Rev. Genet..

[14] Wyatt R.A., Keow J.Y., Harris N.D., Haché C.A., Li D.H., Crawford B.D. (2009). The Zebrafish Embryo: A Powerful Model System for Investigating Matrix Remodeling. Zebrafish.

[15] Bode W., Gomis-Rüth F.X., Stöckler W. (1993). Astacins, Serralysins, Snake Venom and Matrix Metalloproteinases Exhibit Identical Zinc-Binding Environments (HEXXHXXGXXH and Met-Turn) and Topologies and Should Be Grouped into a Common Family, the “Metzincins”. FEBS Lett..

[16] Li K., Tay F.R., Yiu C.K.Y. (2020). The Past, Present and Future Perspectives of Matrix Metalloproteinase Inhibitors. Pharmacol. Ther..

[17] Gimeno A., Beltrán-Debón R., Mulero M., Pujadas G., Garcia-Vallvé S. (2020). Understanding the Variability of the S1′ Pocket to Improve Matrix Metalloproteinase Inhibitor Selectivity Profiles. Drug Discov. Today.

[18] Gall A.L., Ruff M., Kannan R., Cuniasse P., Yiotakis A., Dive V., Rio M.C., Basset P., Moras D. (2001). Crystal Structure of the Stromelysin-3 (MMP-11) Catalytic Domain Complexed with a Phosphinic Inhibitor Mimicking the Transition-State. J. Mol. Biol..

[19] Laronha H., Caldeira J. (2020). Structure and Function of Human Matrix Metalloproteinases. Cells.

[20] Craig V.J., Zhang L., Hagood J.S., Owen C.A. (2015). Matrix Metalloproteinases as Therapeutic Targets for Idiopathic Pulmonary Fibrosis. Am. J. Respir. Cell Mol. Biol..

[21] Li N., Qiu L., Zeng C., Fang Z., Chen S., Song X., Song H., Zhang G. (2021). Bioinformatic Analysis of Differentially Expressed Genes and Pathways in Idiopathic Pulmonary Fibrosis. Ann. Transl. Med..

[22] Checa M., Ruiz V., Montaño M., Velázquez-Cruz R., Selman M., Pardo A. (2008). MMP-1 Polymorphisms and the Risk of Idiopathic Pulmonary Fibrosis. Hum. Genet..

[23] Wan H., Huang X., Cong P., He M., Chen A., Wu T., Dai D., Li W., Gao X., Tian L. (2021). Identification of Hub Genes and Pathways Associated with Idiopathic Pulmonary Fibrosis via Bioinformatics Analysis. Front. Mol. Biosci..

[24] Gabasa M., Arshakyan M., Llorente A., Chuliá-Peris L., Pavelescu I., Xaubet A., Pereda J., Alcaraz J. (2020). Interleukin-1β Modulation of the Mechanobiology of Primary Human Pulmonary Fibroblasts: Potential Implications in Lung Repair. Int. J. Mol. Sci..

[25] Wu B., Crampton S.P., Hughes C.C.W. (2007). Wnt Signaling Induces Matrix Metalloproteinase Expression and Regulates T Cell Transmigration. Immunity.

[26] Xaubet A., Marin-Arguedas A., Lario S., Ancochea J., Morell F., Ruiz-Manzano J., Rodriguez-Becerra E., Rodriguez-Arias J.M., Iñigo P., Sanz S. (2003). Transforming Growth Factor-Β1 Gene Polymorphisms Are Associated with Disease Progression in Idiopathic Pulmonary Fibrosis. Am. J. Respir. Crit. Care Med..

[27] Sueblinvong V., Neveu W.A., Neujahr D.C., Mills S.T., Rojas M., Roman J., Guidot D.M. (2014). Aging Promotes Pro-Fibrotic Matrix Production and Increases Fibrocyte Recruitment during Acute Lung Injury. Adv. Biosci. Biotechnol..

[28] Kim T.H., Kim S.H., Seo J.Y., Chung H., Kwak H.J., Lee S.K., Yoon H.J., Shin D.H., Park S.S., Sohn J.W. (2011). Blockade of the Wnt/β-Catenin Pathway Attenuates Bleomycin-Induced Pulmonary Fibrosis. Tohoku J. Exp. Med..

[29] Yamashita C.M., Dolgonos L., Zemans R.L., Young S.K., Robertson J., Briones N., Suzuki T., Campbell M.N., Gauldie J., Radisky D.C. (2011). Matrix Metalloproteinase 3 Is a Mediator of Pulmonary Fibrosis. Am. J. Pathol..

[30] Maeda S., Dean D.D., Gomez R., Schwartz Z., Boyan B.D. (2002). The First Stage of Transforming Growth Factor Beta1 Activation Is Release of the Large Latent Complex from the Extracellular Matrix of Growth Plate Chondrocytes by Matrix Vesicle Stromelysin-1 (MMP-3). Calcif. Tissue Int..

[31] Heljasvaara R., Nyberg P., Luostarinen J., Parikka M., Heikkilä P., Rehn M., Sorsa T., Salo T., Pihlajaniemi T. (2005). Generation of Biologically Active Endostatin Fragments from Human Collagen XVIII by Distinct Matrix Metalloproteases. Exp. Cell Res..

[32] Ito T.K., Ishii G., Saito S., Yano K., Hoshino A., Suzuki T., Ochiai A. (2009). Degradation of Soluble VEGF Receptor-1 by MMP-7 Allows VEGF Access to Endothelial Cells. Blood.

[33] Zhang Z., Garron T.M., Li X.J., Liu Y., Zhang X., Li Y.Y., Xu W.S. (2009). Recombinant Human Decorin Inhibits TGF-Beta1-Induced Contraction of Collagen Lattice by Hypertrophic Scar Fibroblasts. Burns.

[34] Kristensen J.H., Larsen L., Dasgupta B., Brodmerkel C., Curran M., Karsdal M.A., Sand J.M.B., Willumsen N., Knox A.J., Bolton C.E. (2015). Levels of Circulating MMP-7 Degraded Elastin Are Elevated in Pulmonary Disorders. Clin. Biochem..

[35] Majewski S., Szewczyk K., Żal A., Białas A.J., Miłkowska-Dymanowska J., Piotrowski W.J. (2021). Serial Measurements of Circulating KL-6, SP-D, MMP-7, CA19-9, CA-125, CCL18, and Periostin in Patients with Idiopathic Pulmonary Fibrosis Receiving Antifibrotic Therapy: An Exploratory Study. J. Clin. Med..

[36] García-de-Alba C., Becerril C., Ruiz V., González Y., Reyes S., García-Alvarez J., Selman M., Pardo A. (2010). Expression of Matrix Metalloproteases by Fibrocytes: Possible Role in Migration and Homing. Am. J. Respir. Crit. Care Med..

[37] García-Prieto E., González-López A., Cabrera S., Astudillo A., Gutiérrez-Fernández A., Fanjul-Fernandez M., Batalla-Solís E., Puente X.S., Fueyo A., López-Otín C. (2010). Resistance to Bleomycin-Induced Lung Fibrosis in MMP-8 Deficient Mice Is Mediated by Interleukin-10. PLoS ONE.

[38] Tager A.M., Kradin R.L., Lacamera P., Bercury S.D., Campanella G.S.V., Leary C.P., Polosukhin V., Zhao L.H., Sakamoto H., Blackwell T.S. (2004). Inhibition of Pulmonary Fibrosis by the Chemokine IP-10/CXCL10. Am. J. Respir. Cell Mol. Biol..

[39] Lakatos G., Sipos F., Miheller P., Hritz I., Varga M.Z., Juhász M., Molnár B., Tulassay Z., Herszényi L. (2012). The Behavior of Matrix Metalloproteinase-9 in Lymphocytic Colitis, Collagenous Colitis and Ulcerative Colitis. Pathol. Oncol. Res..

[40] Betsuyaku T., Fukuda Y., Parks W.C., Shipley J.M., Senior R.M. (2000). Gelatinase B Is Required for Alveolar Bronchiolization after Intratracheal Bleomycin. Am. J. Pathol..

[41] Cabrera S., Gaxiola M., Arreola J.L., Ramírez R., Jara P., D’Armiento J., Richards T., Selman M., Pardo A. (2007). Overexpression of MMP9 in Macrophages Attenuates Pulmonary Fibrosis Induced by Bleomycin. Int. J. Biochem. Cell Biol..

[42] Sokai A., Handa T., Tanizawa K., Oga T., Uno K., Tsuruyama T., Kubo T., Ikezoe K., Nakatsuka Y., Tanimura K. (2015). Matrix Metalloproteinase-10: A Novel Biomarker for Idiopathic Pulmonary Fibrosis. Respir. Res..

[43] Rohani M.G., McMahan R.S., Razumova M.V., Hertz A.L., Cieslewicz M., Pun S.H., Regnier M., Wang Y., Birkland T.P., Parks W.C. (2015). MMP-10 Regulates Collagenolytic Activity of Alternatively Activated Resident Macrophages. J. Investig. Dermatol..

[44] Mukhi S., Brown D.D. (2011). Transdifferentiation of Tadpole Pancreatic Acinar Cells to Duct Cells Mediated by Notch and Stromelysin-3. Dev. Biol..

[45] Rooman I., De Medts N., Baeyens L., Lardon J., De Breuck S., Heimberg H., Bouwens L. (2006). Expression of the Notch Signaling Pathway and Effect on Exocrine Cell Proliferation in Adult Rat Pancreas. Am. J. Pathol..

[46] Aoyagi-Ikeda K., Maeno T., Matsui H., Ueno M., Hara K., Aoki Y., Aoki F., Shimizu T., Doi H., Kawai-Kowase K. (2011). Notch Induces Myofibroblast Differentiation of Alveolar Epithelial Cells via Transforming Growth Factor-{beta}-Smad3 Pathway. Am. J. Respir. Cell Mol. Biol..

[47] Kang H.R., Soo J.C., Chun G.L., Homer R.J., Elias J.A. (2007). Transforming Growth Factor (TGF)-Beta1 Stimulates Pulmonary Fibrosis and Inflammation via a Bax-Dependent, Bid-Activated Pathway That Involves Matrix Metalloproteinase-12. J. Biol. Chem..

[48] Guan C., Xiao Y., Li K., Wang T., Liang Y., Liao G. (2019). MMP-12 Regulates Proliferation of Mouse Macrophages via the ERK/P38 MAPK Pathways during Inflammation. Exp. Cell Res..

[49] Matute-Bello G., Wurfel M.M., Lee J.S., Park D.R., Frevert C.W., Madtes D.K., Shapiro S.D., Martin T.R. (2007). Essential Role of MMP-12 in Fas-Induced Lung Fibrosis. Am. J. Respir. Cell Mol. Biol..

[50] Cabrera S., Maciel M., Hernández-Barrientos D., Calyeca J., Gaxiola M., Selman M., Pardo A. (2019). Delayed Resolution of Bleomycin-Induced Pulmonary Fibrosis in Absence of MMP13 (Collagenase 3). Am. J. Physiol. Lung Cell. Mol. Physiol..

[51] Flechsig P., Hartenstein B., Teurich S., Dadrich M., Hauser K., Abdollahi A., Gröne H.J., Angel P., Huber P.E. (2010). Loss of Matrix Metalloproteinase-13 Attenuates Murine Radiation-Induced Pulmonary Fibrosis. Int. J. Radiat. Oncol. Biol. Phys..

[52] Sen A.I., Shiomi T., Okada Y., D’Armiento J.M. (2010). Deficiency of Matrix Metalloproteinase-13 Increases Inflammation after Acute Lung Injury. Exp. Lung Res..

[53] Nkyimbeng T., Ruppert C., Shiomi T., Dahal B., Lang G., Seeger W., Okada Y., D’Armiento J., Günther A. (2013). Pivotal Role of Matrix Metalloproteinase 13 in Extracellular Matrix Turnover in Idiopathic Pulmonary Fibrosis. PLoS ONE.

[54] Xiong Y., Zhang J., Shi L., Ning Y., Zhu Y., Chen S., Yang M., Chen J., Zhou G.-W., Li Q. (2017). NOGO-B Promotes EMT in Lung Fibrosis via MMP14 Mediates Free TGF-Beta1 Formation. Oncotarget.

[55] Gutiérrez-Fernández A., Soria-Valles C., Osorio F.G., Gutiérrez-Abril J., Garabaya C., Aguirre A., Fueyo A., Fernández-García M.S., Puente X.S., López-Otín C. (2015). Loss of MT1-MMP Causes Cell Senescence and Nuclear Defects Which Can Be Reversed by Retinoic Acid. EMBO J..

[56] Placido L., Romero Y., Maldonado M., Toscano-Marquez F., Ramírez R., Calyeca J., Mora A.L., Selman M., Pardo A. (2021). Loss of MT1-MMP in Alveolar Epithelial Cells Exacerbates Pulmonary Fibrosis. Int. J. Mol. Sci..

[57] López B., González A., Hermida N., Valencia F., De Teresa E., Díez J. (2010). Role of Lysyl Oxidase in Myocardial Fibrosis: From Basic Science to Clinical Aspects. Am. J. Physiol. Heart Circ. Physiol..

[58] Yu G., Kovkarova-Naumovski E., Jara P., Parwani A., Kass D., Ruiz V., Lopez-Otiń C., Rosas I.O., Gibson K.F., Cabrera S. (2012). Matrix Metalloproteinase-19 Is a Key Regulator of Lung Fibrosis in Mice and Humans. Am. J. Respir. Crit. Care Med..

[59] Jara P., Calyeca J., Romero Y., Plácido L., Yu G., Kaminski N., Maldonado V., Cisneros J., Selman M., Pardo A. (2015). Matrix Metalloproteinase (MMP)-19-Deficient Fibroblasts Display a Profibrotic Phenotype. Am. J. Physiol. Lung Cell. Mol. Physiol..

[60] Illman S.A., Keski-Oja J., Parks W.C., Lohi J. (2003). The Mouse Matrix Metalloproteinase, Epilysin (MMP-28), Is Alternatively Spliced and Processed by a Furin-like Proprotein Convertase. Biochem. J..

[61] Manicone A.M., Birkland T.P., Lin M., Betsuyaku T., van Rooijen N., Lohi J., Keski-Oja J., Wang Y., Skerrett S.J., Parks W.C. (2009). Epilysin (MMP-28) Restrains Early Macrophage Recruitment in Pseudomonas Aeruginosa Pneumonia. J. Immunol..

[62] Gharib S.A., Johnston L.K., Huizar I., Birkland T.P., Hanson J., Wang Y., Parks W.C., Manicone A.M. (2014). MMP28 Promotes Macrophage Polarization toward M2 Cells and Augments Pulmonary Fibrosis. J. Leukoc. Biol..

[63] Maldonado M., Buendía-Roldán I., Vicens-Zygmunt V., Planas L., Molina-Molina M., Selman M., Pardo A. (2018). Identification of MMP28 as a Biomarker for the Differential Diagnosis of Idiopathic Pulmonary Fibrosis. PLoS ONE.

[64] Yan C., Boyd D.D. (2007). Regulation of Matrix Metalloproteinase Gene Expression. J. Cell. Physiol..

[65] Overall C.M., Wrana J.L., Sodek J. (1991). Transcriptional and Post-Transcriptional Regulation of 72-KDa Gelatinase/Type IV Collagenase by Transforming Growth Factor-Beta 1 in Human Fibroblasts. Comparisons with Collagenase and Tissue Inhibitor of Matrix Metalloproteinase Gene Expression. J. Biol. Chem..

[66] Saarialho-Kere U.K., Welgus H.G., Parks W.C. (1993). Distinct Mechanisms Regulate Interstitial Collagenase and 92-KDa Gelatinase Expression in Human Monocytic-like Cells Exposed to Bacterial Endotoxin. J. Biol. Chem..

[67] Shi C., Wu L., Lin W., Cai Y., Zhang Y., Hu B., Gao R., Im H.J., Yuan W., Ye X. (2019). MiR-202-3p Regulates Interleukin-1β-Induced Expression of Matrix Metalloproteinase 1 in Human Nucleus Pulposus. Gene.

[68] Liang Z.J., Zhuang H., Wang G.X., Li Z., Zhang H.T., Yu T.Q., Zhang B.D. (2012). MiRNA-140 Is a Negative Feedback Regulator of MMP-13 in IL-1β-Stimulated Human Articular Chondrocyte C28/I2 Cells. Inflamm. Res..

[69] Lou L., Tian M., Chang J., Li F., Zhang G. (2020). MiRNA-192-5p Attenuates Airway Remodeling and Autophagy in Asthma by Targeting MMP-16 and ATG7. Biomed. Pharmacother..

[70] Rak B., Mehlich D., Garbicz F., Domosud Z., Paskal W., Marczewska J.M., Włodarski P.K. (2017). Post-Transcriptional Regulation of MMP16 and TIMP2 Expression via MiR-382, MiR-410 and MiR-200b in Endometrial Cancer. Cancer Genom. Proteom..

[71] Asuthkar S., Velpula K.K., Chetty C., Gorantla B., Rao J.S., Asuthkar S., Velpula K.K., Chetty C., Gorantla B., Rao J.S. (2012). Epigenetic Regulation of MiRNA-211 by MMP-9 Governs Glioma Cell Apoptosis, Chemosensitivity and Radiosensitivity. Oncotarget.

[72] Van Wart H.E., Birkedal-Hansen H. (1990). The Cysteine Switch: A Principle of Regulation of Metalloproteinase Activity with Potential Applicability to the Entire Matrix Metalloproteinase Gene Family. Proc. Natl. Acad. Sci. USA.

[73] Fallata A.M., Wyatt R.A., Levesque J.M., Dufour A., Overall C.M., Crawford B.D. (2019). Intracellular Localization in Zebrafish Muscle and Conserved Sequence Features Suggest Roles for Gelatinase A Moonlighting in Sarcomere Maintenance. Biomedicines.

[74] Hey S., Ratt A., Linder S. (2022). There and Back Again: Intracellular Trafficking, Release and Recycling of Matrix Metalloproteinases. Biochim. Biophys. Acta Mol. Cell Res..

[75] Etienne-Manneville S. (2013). Microtubules in Cell Migration. Annu. Rev. Cell Dev. Biol..

[76] Rottner K., Faix J., Bogdan S., Linder S., Kerkhoff E. (2017). Actin Assembly Mechanisms at a Glance. J. Cell Sci..

[77] Linder S. (2007). The Matrix Corroded: Podosomes and Invadopodia in Extracellular Matrix Degradation. Trends Cell Biol..

[78] Owen C.A., Hu Z., Lopez-Otin C., Shapiro S.D. (2004). Membrane-Bound Matrix Metalloproteinase-8 on Activated Polymorphonuclear Cells Is a Potent, Tissue Inhibitor of Metalloproteinase-Resistant Collagenase and Serpinase. J. Immunol..

[79] Koo B.H., Kim H.H., Park M.Y., Jeon O.H., Kim D.S. (2009). Membrane Type-1 Matrix Metalloprotease-Independent Activation of pro-Matrix Metalloprotease-2 by Proprotein Convertases. FEBS J..

[80] Nagase H., Visse R., Murphy G. (2006). Structure and Function of Matrix Metalloproteinases and TIMPs. Cardiovasc. Res..

[81] Cabral-Pacheco G.A., Garza-Veloz I., Castruita-De la Rosa C., Ramirez-Acuña J.M., Perez-Romero B.A., Guerrero-Rodriguez J.F., Martinez-Avila N., Martinez-Fierro M.L. (2020). The Roles of Matrix Metalloproteinases and Their Inhibitors in Human Diseases. Int. J. Mol. Sci..

[82] Yu W.H., Yu S.S.C., Meng Q., Brew K., Woessner J.F. (2000). TIMP-3 Binds to Sulfated Glycosaminoglycans of the Extracellular Matrix. J. Biol. Chem..

[83] Majali-Martinez A., Hiden U., Ghaffari-Tabrizi-Wizsy N., Lang U., Desoye G., Dieber-Rotheneder M. (2016). Placental Membrane-Type Metalloproteinases (MT-MMPs): Key Players in Pregnancy. Cell Adh. Migr..

[84] Morgunova E., Tuuttila A., Bergmann U., Tryggvason K. (2002). Structural Insight into the Complex Formation of Latent Matrix Metalloproteinase 2 with Tissue Inhibitor of Metalloproteinase 2. Proc. Natl. Acad. Sci. USA.

[85] Matchett E.F., Wang S., Crawford B.D. (2019). Paralogues of Mmp11 and Timp4 Interact during the Development of the Myotendinous Junction in the Zebrafish Embryo. J. Dev. Biol..

[86] Giannandrea M., Parks W.C. (2014). Diverse Functions of Matrix Metalloproteinases during Fibrosis. Dis. Model. Mech..

[87] Egeblad M., Werb Z. (2002). New Functions for the Matrix Metalloproteinases in Cancer Progression. Nat. Rev. Cancer.

[88] Dumin J.A., Dickeson S.K., Stricker T.P., Bhattacharyya-Pakrasi M., Roby J.D., Santoro S.A., Parks W.C. (2001). Pro-Collagenase-1 (Matrix Metalloproteinase-1) Binds the Alpha(2)Beta(1) Integrin upon Release from Keratinocytes Migrating on Type I Collagen. J. Biol. Chem..

[89] Riikonen T., Westermarck J., Koivisto L., Broberg A., Kahari V.M., Heino J. (1995). Integrin Alpha 2 Beta 1 Is a Positive Regulator of Collagenase (MMP-1) and Collagen Alpha 1(I) Gene Expression. J. Biol. Chem..

[90] Suzuki K., Morodomi T., Nagase H., Enghild J.J., Salvesen G. (1990). Mechanisms of Activation of Tissue Procollagenase by Matrix Metalloproteinase 3 (Stromelysin). Biochemistry.

[91] Rosas I.O., Richards T.J., Konishi K., Zhang Y., Gibson K., Lokshin A.E., Lindell K.O., Cisneros J., MacDonald S.D., Pardo A. (2008). MMP1 and MMP7 as Potential Peripheral Blood Biomarkers in Idiopathic Pulmonary Fibrosis. PLoS Med..

[92] Vij R., Noth I. (2012). Peripheral Blood Biomarkers in Idiopathic Pulmonary Fibrosis. Transl. Res..

[93] Selman M., Ruiz V., Cabrera S., Segura L., Ramírez R., Barrios R., Pardo A. (2000). TIMP-1, -2, -3, and -4 in Idiopathic Pulmonary Fibrosis. A Prevailing Nondegradative Lung Microenvironment?. Am. J. Physiol. Lung Cell. Mol. Physiol..

[94] Herrera I., Cisneros J., Maldonado M., Ramírez R., Ortiz-Quintero B., Anso E., Chandel N.S., Selman M., Pardo A. (2013). Matrix Metalloproteinase (MMP)-1 Induces Lung Alveolar Epithelial Cell Migration and Proliferation, Protects from Apoptosis, and Represses Mitochondrial Oxygen Consumption. J. Biol. Chem..

[95] Weng T., Poth J.M., Karmouty-Quintana H., Garcia-Morales L.J., Melicoff E., Luo F., Chen N.Y., Evans C.M., Bunge R.R., Bruckner B.A. (2014). Hypoxia-Induced Deoxycytidine Kinase Contributes to Epithelial Proliferation in Pulmonary Fibrosis. Am. J. Respir. Crit. Care Med..

[96] Estany S., Vicens-Zygmunt V., Llatjós R., Montes A., Penín R., Escobar I., Xaubet A., Santos S., Manresa F., Dorca J. (2014). Lung Fibrotic Tenascin-C Upregulation Is Associated with Other Extracellular Matrix Proteins and Induced by TGFβ1. BMC Pulm. Med..

[97] Konishi K., Gibson K.F., Lindell K.O., Richards T.J., Zhang Y., Dhir R., Bisceglia M., Gilbert S., Yousem S.A., Jin W.S. (2009). Gene Expression Profiles of Acute Exacerbations of Idiopathic Pulmonary Fibrosis. Am. J. Respir. Crit. Care Med..

[98] Zuo F., Kaminski N., Eugui E., Allard J., Yakhini Z., Ben-Dor A., Lollini L., Morris D., Kim Y., DeLustro B. (2002). Gene Expression Analysis Reveals Matrilysin as a Key Regulator of Pulmonary Fibrosis in Mice and Humans. Proc. Natl. Acad. Sci. USA.

[99] Kreus M., Lehtonen S., Skarp S., Kaarteenaho R. (2021). Extracellular Matrix Proteins Produced by Stromal Cells in Idiopathic Pulmonary Fibrosis and Lung Adenocarcinoma. PLoS ONE.

[100] Roach K.M., Castells E., Dixon K., Mason S., Elliott G., Marshall H., Poblocka M.A., Macip S., Richardson M., Khalfaoui L. (2021). Evaluation of Pirfenidone and Nintedanib in a Human Lung Model of Fibrogenesis. Front. Pharmacol..

[101] Gabasa M., Radisky E.S., Ikemori R., Bertolini G., Arshakyan M., Hockla A., Duch P., Rondinone O., Llorente A., Maqueda M. (2021). MMP1 Drives Tumor Progression in Large Cell Carcinoma of the Lung through Fibroblast Senescence. Cancer Lett..

[102] Vincenti M.P., Brinckerhoff C.E. (2002). Transcriptional Regulation of Collagenase (MMP-1, MMP-13) Genes in Arthritis: Integration of Complex Signaling Pathways for the Recruitment of Gene-Specific Transcription Factors. Arthritis Res..

[103] Segura-Valdez L., Pardo A., Gaxiola M., Uhal B.D., Becerril C., Selman M. (2000). Upregulation of Gelatinases A and B, Collagenases 1 and 2, and Increased Parenchymal Cell Death in COPD. Chest.

[104] Imai K., Dalal S.S., Chen E.S., Downey R., Schulman L.L., Ginsburg M., D’Armiento J. (2001). Human Collagenase (Matrix Metalloproteinase-1) Expression in the Lungs of Patients with Emphysema. Am. J. Respir. Crit. Care Med..

[105] Mercer B.A., Kolesnikova N., Sonett J., D’Armiento J. (2004). Extracellular Regulated Kinase/Mitogen Activated Protein Kinase Is up-Regulated in Pulmonary Emphysema and Mediates Matrix Metalloproteinase-1 Induction by Cigarette Smoke. J. Biol. Chem..

[106] Pardo A., Cabrera S., Maldonado M., Selman M. (2016). Role of Matrix Metalloproteinases in the Pathogenesis of Idiopathic Pulmonary Fibrosis. Respir. Res..

[107] Strongin A.Y., Collier I., Bannikov G., Marmer B.L., Grant G.A., Goldberg G.I. (1995). Mechanism of Cell Surface Activation of 72-KDa Type IV Collagenase. Isolation of the Activated Form of the Membrane Metalloprotease. J. Biol. Chem..

[108] Sato H., Takino T., Okada Y., Cao J., Shinagawa A., Yamamoto E., Seiki M. (1994). A Matrix Metalloproteinase Expressed on the Surface of Invasive Tumour Cells. Nature.

[109] Owen C.A. (2008). Leukocyte Cell Surface Proteinases: Regulation of Expression, Functions, and Mechanisms of Surface Localization. Int. J. Biochem. Cell Biol..

[110] Chun H. (1996). Molecular Mechanism of Transcriptional Activation of Human Gelatinase B by Proximal Promoter. Cancer Lett..

[111] Hrabec E., Strek M., Nowak D., Greger J., Suwalski M., Hrabec Z. (2002). Activity of Type IV Collagenases (MMP-2 and MMP-9) in Primary Pulmonary Carcinomas: A Quantitative Analysis. J. Cancer Res. Clin. Oncol..

[112] Chakraborti S., Mandal M., Das S., Mandal A., Chakraborti T. (2003). Regulation of Matrix Metalloproteinases: An Overview. Mol. Cell. Biochem..

[113] Rojas M., Mora A.L., Kapetanaki M., Weathington N., Gladwin M., Eickelberg O. (2015). Aging and Lung Disease. Clinical Impact and Cellular and Molecular Pathways. Ann. Am. Thorac. Soc..

[114] Ruiz V., Ordóñez R.M., Berumen J., Ramírez R., Uhal B., Becerril C., Pardo A., Selman M. (2003). Unbalanced Collagenases/TIMP-1 Expression and Epithelial Apoptosis in Experimental Lung Fibrosis. Am. J. Physiol. Lung Cell. Mol. Physiol..

[115] Keane M.P., Belperio J.A., Burdick M.D., Lynch J.P., Fishbein M.C., Strieter R.M. (2001). ENA-78 Is an Important Angiogenic Factor in Idiopathic Pulmonary Fibrosis. Am. J. Respir. Crit. Care Med..

[116] Bergers G., Brekken R., McMahon G., Vu T.H., Itoh T., Tamaki K., Tanzawa K., Thorpe P., Itohara S., Werb Z. (2000). Matrix Metalloproteinase-9 Triggers the Angiogenic Switch during Carcinogenesis. Nat. Cell Biol..

[117] Nguyen M., Arkell J., Jackson C.J. (2001). Human Endothelial Gelatinases and Angiogenesis. Int. J. Biochem. Cell Biol..

[118] Narumiya H., Zhang Y., Fernandez-Patron C., Guilbert L.J., Davidge S.T. (2001). Matrix Metalloproteinase-2 Is Elevated in the Plasma of Women with Preeclampsia. Hypertens. Pregnancy.

[119] Hamada N., Kuwano K., Yamada M., Hagimoto N., Hiasa K., Egashira K., Nakashima N., Maeyama T., Yoshimi M., Nakanishi Y. (2005). Anti-Vascular Endothelial Growth Factor Gene Therapy Attenuates Lung Injury and Fibrosis in Mice. J. Immunol..

[120] Chetty C., Lakka S.S., Bhoopathi P., Rao J.S. (2010). MMP-2 Alters VEGF Expression via AVβ3 Integrin-Mediated PI3K/AKT Signaling in A549 Lung Cancer Cells. Int. J. Cancer.

[121] Li L., Li Q., Wei L., Wang Z., Ma W., Liu F., Shen Y., Zhang S., Zhang X., Li H. (2019). Chemokine (C-X-C Motif) Ligand 14 Contributes to Lipopolysaccharide-Induced Fibrogenesis in Mouse L929 Fibroblasts via Modulating PPM1A. J. Cell. Biochem..

[122] Lam A.P., Herazo-Maya J.D., Sennello J.A., Flozak A.S., Russell S., Mutlu G.M., Budinger G.R.S., DasGupta R., Varga J., Kaminski N. (2014). Wnt Coreceptor Lrp5 Is a Driver of Idiopathic Pulmonary Fibrosis. Am. J. Respir. Crit. Care Med..

[123] Habgood A.N., Tatler A.L., Porte J., Wahl S.M., Laurent G.J., John A.E., Johnson S.R., Jenkins G. (2016). Secretory Leukocyte Protease Inhibitor Gene Deletion Alters Bleomycin-Induced Lung Injury, but Not Development of Pulmonary Fibrosis. Lab. Investig..

[124] Xie B., Zheng G., Li H., Yao X., Hong R., Li R., Yue W., Chen Y. (2016). Effects of the Tumor Suppressor PTEN on the Pathogenesis of Idiopathic Pulmonary Fibrosis in Chinese Patients. Mol. Med. Rep..

[125] McKeown S., Richter A.G., O’Kane C., McAuley D.F., Thickett D.R. (2009). MMP Expression and Abnormal Lung Permeability Are Important Determinants of Outcome in IPF. Eur. Respir. J..

[126] Selman M., Pardo A. (2006). Role of Epithelial Cells in Idiopathic Pulmonary Fibrosis: From Innocent Targets to Serial Killers. Proc. Am. Thorac. Soc..

[127] Richter A.G., McKeown S., Rathinam S., Harper L., Rajesh P., McAuley D.F., Heljasvaara R., Thickett D.R. (2009). Soluble Endostatin Is a Novel Inhibitor of Epithelial Repair in Idiopathic Pulmonary Fibrosis. Thorax.

[128] Dancer R.C.A., Wood A.M., Thickett D.R. (2011). Metalloproteinases in Idiopathic Pulmonary Fibrosis. Eur. Respir. J..

[129] Pardo A., Gibson K., Cisneros J., Richards T.J., Yang Y., Becerril C., Yousem S., Herrera I., Ruiz V., Selman M. (2005). Up-Regulation and Profibrotic Role of Osteopontin in Human Idiopathic Pulmonary Fibrosis. PLoS Med..

[130] Agnihotri R., Crawford H.C., Haro H., Matrisian L.M., Havrda M.C., Liaw L. (2001). Osteopontin, a Novel Substrate for Matrix Metalloproteinase-3 (Stromelysin-1) and Matrix Metalloproteinase-7 (Matrilysin). J. Biol. Chem..

[131] Li Q., Park P.W., Wilson C.L., Parks W.C. (2002). Matrilysin Shedding of Syndecan-1 Regulates Chemokine Mobilization and Transepithelial Efflux of Neutrophils in Acute Lung Injury. Cell.

[132] Manicone A.M., Huizar I., McGuire J.K. (2009). Matrilysin (Matrix Metalloproteinase-7) Regulates Anti-Inflammatory and Antifibrotic Pulmonary Dendritic Cells That Express CD103 (Alpha(E)Beta(7)-Integrin). Am. J. Pathol..

[133] Imai K., Hiramatsu A., Fukushima D., Pierschbacher M.D., Okada Y. (1997). Degradation of Decorin by Matrix Metalloproteinases: Identification of the Cleavage Sites, Kinetic Analyses and Transforming Growth Factor-Beta1 Release. Biochem. J..

[134] Schönherr E., Broszat M., Brandan E., Bruckner P., Kresse H. (1998). Decorin Core Protein Fragment Leu155-Val260 Interacts with TGF-Beta but Does Not Compete for Decorin Binding to Type I Collagen. Arch. Biochem. Biophys..

[135] Blobe G.C., Schiemann W.P., Lodish H.F. (2000). Role of Transforming Growth Factor Beta in Human Disease. N. Engl. J. Med..

[136] Bauer Y., White E.S., de Bernard S., Cornelisse P., Leconte I., Morganti A., Roux S., Nayler O. (2017). MMP-7 Is a Predictive Biomarker of Disease Progression in Patients with Idiopathic Pulmonary Fibrosis. ERJ Open Res..

[137] Tzouvelekis A., Herazo-Maya J.D., Slade M., Chu J.H., Deiuliis G., Ryu C., Li Q., Sakamoto K., Ibarra G., Pan H. (2017). Validation of the Prognostic Value of MMP-7 in Idiopathic Pulmonary Fibrosis. Respirology.

[138] Cabrera Cesar E., Lopez-Lopez L., Lara E., Hidalgo-San Juan M.V., Parrado Romero C., Palencia J.L.R.S., Martín-Montañez E., Garcia-Fernandez M. (2021). Serum Biomarkers in Differential Diagnosis of Idiopathic Pulmonary Fibrosis and Connective Tissue Disease-Associated Interstitial Lung Disease. J. Clin. Med..

[139] Clynick B., Corte T.J., Jo H.E., Stewart I., Glaspole I.N., Grainge C., Maher T.M., Navaratnam V., Hubbard R., Hopkins P.M.A. (2021). Biomarker Signatures for Progressive Idiopathic Pulmonary Fibrosis. Eur. Respir. J..

[140] Konigsberg I.R., Borie R., Walts A.D., Cardwell J., Rojas M., Metzger F., Hauck S.M., Fingerlin T.E., Yang I.V., Schwartz D.A. (2021). Molecular Signatures of Idiopathic Pulmonary Fibrosis. Am. J. Respir. Cell Mol. Biol..

[141] Kalafatis D., Löfdahl A., Näsman P., Dellgren G., Wheelock Å.M., Elowsson Rendin L., Sköld M., Westergren-Thorsson G. (2021). Distal Lung Microenvironment Triggers Release of Mediators Recognized as Potential Systemic Biomarkers for Idiopathic Pulmonary Fibrosis. Int. J. Mol. Sci..

[142] Khan F.A., Stewart I., Saini G., Robinson K.A., Jenkins R.G. (2021). A Systematic Review of Blood Biomarkers with Individual Participant Data Meta-Analysis of Matrix-Metalloproteinase-7 in IPF. Eur. Respir. J..

[143] Anacker J., Segerer S.E., Hagemann C., Feix S., Kapp M., Bausch R., Kämmerer U. (2011). Human Decidua and Invasive Trophoblasts Are Rich Sources of Nearly All Human Matrix Metalloproteinases. Mol. Hum. Reprod..

[144] Chapoval S.P., Lee C.G., Tang C., Keegan A.D., Cohn L., Bottomly K., Elias J.A. (2009). Lung Vascular Endothelial Growth Factor Expression Induces Local Myeloid Dendritic Cell Activation. Clin. Immunol..

[145] Akhavani M.A., Madden L., Buysschaert I., Sivakumar B., Kang N., Paleolog E.M. (2009). Hypoxia Upregulates Angiogenesis and Synovial Cell Migration in Rheumatoid Arthritis. Arthritis Res. Ther..

[146] Craig V.J., Quintero P.A., Fyfe S.E., Patel A.S., Knolle M.D., Kobzik L., Owen C.A. (2013). Profibrotic Activities for Matrix Metalloproteinase-8 during Bleomycin-Mediated Lung Injury. J. Immunol..

[147] Hanemaaijer R., Sorsa T., Konttinen Y.T., Ding Y., Sutinen M., Visser H., Van Hinsbergh V.W.M., Helaakoski T., Kainulainen T., Rönkä H. (1997). Matrix Metalloproteinase-8 Is Expressed in Rheumatoid Synovial Fibroblasts and Endothelial Cells. Regulation by Tumor Necrosis Factor-Alpha and Doxycycline. J. Biol. Chem..

[148] Herman M.P., Sukhova G.K., Libby P., Gerdes N., Tang N., Horton D.B., Kilbride M., Breitbart R.E., Chun M., Schönbeck U. (2001). Expression of Neutrophil Collagenase (Matrix Metalloproteinase-8) in Human Atheroma: A Novel Collagenolytic Pathway Suggested by Transcriptional Profiling. Circulation.

[149] Craig V.J., Polverino F., Laucho-Contreras M.E., Shi Y., Liu Y., Osorio J.C., Tesfaigzi Y., Pinto-Plata V., Gochuico B.R., Rosas I.O. (2014). Mononuclear Phagocytes and Airway Epithelial Cells: Novel Sources of Matrix Metalloproteinase-8 (MMP-8) in Patients with Idiopathic Pulmonary Fibrosis. PLoS ONE.

[150] Henry M.T., McMahon K., Mackarel A.J., Prikk K., Sorsa T., Maisi P., Sepper R., FitzGerald M.X., O’Connor C.M. (2002). Matrix Metalloproteinases and Tissue Inhibitor of Metalloproteinase-1 in Sarcoidosis and IPF. Eur. Respir. J..

[151] Stijn W., Verleden Stijn E., Vanaudenaerde Bart M., Marijke W., Christophe D., Jonas Y., Jana S., Verbeken Eric K., Verleden Geert M., Wuyts Wim A. (2013). Multiplex Protein Profiling of Bronchoalveolar Lavage in Idiopathic Pulmonary Fibrosis and Hypersensitivity Pneumonitis. Ann. Thorac. Med..

[152] Todd J.L., Vinisko R., Liu Y., Neely M.L., Overton R., Flaherty K.R., Noth I., Newby L.K., Lasky J.A., Olman M.A. (2020). Circulating Matrix Metalloproteinases and Tissue Metalloproteinase Inhibitors in Patients with Idiopathic Pulmonary Fibrosis in the Multicenter IPF-PRO Registry Cohort. BMC Pulm. Med..

[153] Sun L., Louie M.C., Vannella K.M., Wilke C.A., Levine A.M., Moore B.B., Shanley T.P. (2011). New Concepts of IL-10-Induced Lung Fibrosis: Fibrocyte Recruitment and M2 Activation in a CCL2/CCR2 Axis. Am. J. Physiol. Lung Cell. Mol. Physiol..

[154] Suga M., Iyonaga K., Okamoto T., Gushima Y., Miyakawa H., Akaike T., Ando M. (2000). Characteristic Elevation of Matrix Metalloproteinase Activity in Idiopathic Interstitial Pneumonias. Am. J. Respir. Crit. Care Med..

[155] Lemjabbar H., Gosset P., Lechapt-Zalcman E., Franco-Montoya M.L., Wallaert B., Harf A., Lafuma C. (1999). Overexpression of Alveolar Macrophage Gelatinase B (MMP-9) in Patients with Idiopathic Pulmonary Fibrosis: Effects of Steroid and Immunosuppressive Treatment. Am. J. Respir. Cell Mol. Biol..

[156] Ram M., Sherer Y., Shoenfeld Y. (2006). Matrix Metalloproteinase-9 and Autoimmune Diseases. J. Clin. Immunol..

[157] Overall C.M., López-Otín C. (2002). Strategies for MMP Inhibition in Cancer: Innovations for the Post-Trial Era. Nat. Rev. Cancer.

[158] Peterson J.T. (2006). The Importance of Estimating the Therapeutic Index in the Development of Matrix Metalloproteinase Inhibitors. Cardiovasc. Res..

[159] Gao Q., Meijer M.J.W., Kubben F.J.G.M., Sier C.F.M., Kruidenier L., van Duijn W., van den Berg M., van Hogezand R.A., Lamers C.B.H.W., Verspaget H.W. (2005). Expression of Matrix Metalloproteinases-2 and -9 in Intestinal Tissue of Patients with Inflammatory Bowel Diseases. Dig. Liver Dis..

[160] Gallea-Robache S., Morand V., Millet S., Bruneau J.M., Bhatnagar N., Chouaib S., Roman-Roman S. (1997). A Metalloproteinase Inhibitor Blocks the Shedding of Soluble Cytokine Receptors and Processing of Transmembrane Cytokine Precursors in Human Monocytic Cells. Cytokine.

[161] Odajima N., Betsuyaku T., Nasuhara Y., Nishimura M. (2007). Loss of Caveolin-1 in Bronchiolization in Lung Fibrosis. J. Histochem. Cytochem..

[162] Kawamoto M., Fukuda Y. (1990). Cell Proliferation during the Process of Bleomycin-Induced Pulmonary Fibrosis in Rats. Acta Pathol. Jpn..

[163] Collins J.F., Orozco C.R., McCullough B., Coalson J.J., Johanson W.G. (1982). Pulmonary Fibrosis with Small-Airway Disease: A Model in Nonhuman Primates. Exp. Lung Res..

[164] Fukuda Y., Takemura T., Ferrans V.J. (1989). Evolution of Metaplastic Squamous Cells of Alveolar Walls in Pulmonary Fibrosis Produced by Paraquat. An Ultrastructural and Immunohistochemical Study. Virchows Arch. B Cell Pathol. Incl. Mol. Pathol..

[165] Molyneaux P.L., Willis-Owen S.A.G., Cox M.J., James P., Cowman S., Loebinger M., Blanchard A., Edwards L.M., Stock C., Daccord C. (2017). Host-Microbial Interactions in Idiopathic Pulmonary Fibrosis. Am. J. Respir. Crit. Care Med..

[166] Xu L., Bian W., Gu X.H., Shen C. (2017). Genetic Polymorphism in Matrix Metalloproteinase-9 and Transforming Growth Factor-Β1 and Susceptibility to Combined Pulmonary Fibrosis and Emphysema in a Chinese Population. Kaohsiung J. Med. Sci..

[167] Dayer C., Stamenkovic I. (2015). Recruitment of Matrix Metalloproteinase-9 (MMP-9) to the Fibroblast Cell Surface by Lysyl Hydroxylase 3 (LH3) Triggers Transforming Growth Factor-β (TGF-β) Activation and Fibroblast Differentiation. J. Biol. Chem..

[168] Perng D.W., Chang K.T., Su K.C., Wu Y.C., Chen C.S., Hsu W.H., Tsai C.M., Lee Y.C. (2011). Matrix Metalloprotease-9 Induces Transforming Growth Factor-β(1) Production in Airway Epithelium via Activation of Epidermal Growth Factor Receptors. Life Sci..

[169] Pardo A., Selman M., Kaminski N. (2008). Approaching the Degradome in Idiopathic Pulmonary Fibrosis. Int. J. Biochem. Cell Biol..

[170] Espindola M.S., Habiel D.M., Coelho A.L., Stripp B., Parks W.C., Oldham J., Martinez F.J., Noth I., Lopez D., Mikels-Vigdal A. (2021). Differential Responses to Targeting Matrix Metalloproteinase 9 in Idiopathic Pulmonary Fibrosis. Am. J. Respir. Crit. Care Med..

[171] Ma J.Y., Mercer R.R., Barger M., Schwegler-Berry D., Scabilloni J., Ma J.K., Castranova V. (2012). Induction of Pulmonary Fibrosis by Cerium Oxide Nanoparticles. Toxicol. Appl. Pharmacol..

[172] Scabilloni J.F., Wang L., Antonini J.M., Roberts J.R., Castranova V., Mercer R.R. (2005). Matrix Metalloproteinase Induction in Fibrosis and Fibrotic Nodule Formation Due to Silica Inhalation. Am. J. Physiol. Lung Cell. Mol. Physiol..

[173] Krampert M., Bloch W., Sasaki T., Bugnon P., Rülicke T., Wolf E., Aumailley M., Parks W.C., Werner S. (2004). Activities of the Matrix Metalloproteinase Stromelysin-2 (MMP-10) in Matrix Degradation and Keratinocyte Organization in Wounded Skin. Mol. Biol. Cell.

[174] Garcia-Irigoyen O., Carotti S., Latasa M.U., Uriarte I., Fernández-Barrena M.G., Elizalde M., Urtasun R., Vespasiani-Gentilucci U., Morini S., Banales J.M. (2014). Matrix Metalloproteinase-10 Expression Is Induced during Hepatic Injury and Plays a Fundamental Role in Liver Tissue Repair. Liver Int..

[175] Koller F.L., Dozier E.A., Nam K.T., Swee M., Birkland T.P., Parks W.C., Fingleton B. (2012). Lack of MMP10 Exacerbates Experimental Colitis and Promotes Development of Inflammation-Associated Colonic Dysplasia. Lab. Investig..

[176] Nakamura H., Fujii Y., Ohuchi E., Yamamoto E., Okada Y. (1998). Activation of the Precursor of Human Stromelysin 2 and Its Interactions with Other Matrix Metalloproteinases. Eur. J. Biochem..

[177] Choi M., Cho W.S., Han B.S., Cho M., Kim S.Y., Yi J.Y., Ahn B., Kim S.H., Jeong J. (2008). Transient Pulmonary Fibrogenic Effect Induced by Intratracheal Instillation of Ultrafine Amorphous Silica in A/J Mice. Toxicol. Lett..

[178] Ishikawa F., Miyoshi H., Nose K., Shibanuma M. (2009). Transcriptional Induction of MMP-10 by TGF-β, Mediated by Activation of MEF2A and Downregulation of Class IIa HDACs. Oncogene.

[179] Murray M.Y., Birkland T.P., Howe J.D., Rowan A.D., Fidock M., Parks W.C., Gavrilovic J. (2013). Macrophage Migration and Invasion Is Regulated by MMP10 Expression. PLoS ONE.

[180] Pei D., Weiss S.J. (1995). Furin-Dependent Intracellular Activation of the Human Stromelysin-3 Zymogen. Nature.

[181] Basset P., Bellocq J.P., Wolf C., Stoll I., Hutin P., Limacher J.M., Podhajcer O.L., Chenard M.P., Rio M.C., Chambon P. (1990). A Novel Metalloproteinase Gene Specifically Expressed in Stromal Cells of Breast Carcinomas. Nature.

[182] Belaaouaj A., Shipley J.M., Kobayashi D.K., Zimonjic D.B., Popescu N., Silverman G.A., Shapiro S.D. (1995). Human Macrophage Metalloelastase. Genomic Organization, Chromosomal Location, Gene Linkage, and Tissue-Specific Expression. J. Biol. Chem..

[183] Qu P., Du H., Wang X., Yan C. (2009). Matrix Metalloproteinase 12 Overexpression in Lung Epithelial Cells Plays a Key Role in Emphysema to Lung Bronchioalveolar Adenocarcinoma Transition. Cancer Res..

[184] Brusselle G.G. (2009). Matrix Metalloproteinase 12, Asthma, and COPD. N. Engl. J. Med..

[185] Chen Y.E. (2004). MMP-12, an Old Enzyme Plays a New Role in the Pathogenesis of Rheumatoid Arthritis?. Am. J. Pathol..

[186] Ramalingam T.R., Gieseck R.L., Acciani T.H., Hart K.M., Cheever A.W., Mentink-Kane M.M., Vannella K.M., Wynn T.A. (2016). Enhanced Protection from Fibrosis and Inflammation in the Combined Absence of IL-13 and IFN-γ. J. Pathol..

[187] Sand J.M., Larsen L., Hogaboam C., Martinez F., Han M.L., Larsen M.R., Nawrocki A., Zheng Q., Karsdal M.A., Leeming D.J. (2013). MMP Mediated Degradation of Type IV Collagen Alpha 1 and Alpha 3 Chains Reflects Basement Membrane Remodeling in Experimental and Clinical Fibrosis—Validation of Two Novel Biomarker Assays. PLoS ONE.

[188] Granata S., Santoro G., Masola V., Tomei P., Sallustio F., Pontrelli P., Accetturo M., Antonucci N., Carratú P., Lupo A. (2018). In Vitro Identification of New Transcriptomic and MiRNomic Profiles Associated with Pulmonary Fibrosis Induced by High Doses Everolimus: Looking for New Pathogenetic Markers and Therapeutic Targets. Int. J. Mol. Sci..

[189] Hu B., Wu Z., Bai D., Tang R., Phan S. (2015). Matrix Metalloproteinase-12 (MMP12) Inhibits Myofibroblast Differentiation and Lung Fibrosis. FASEB J..

[190] Ortiz L.A., Lasky J., Gozal E., Ruiz V., Lungarella G., Cavarra E., Brody A.R., Friedman M., Pardo A., Selman M. (2012). Tumor Necrosis Factor Receptor Deficiency Alters Matrix Metalloproteinase 13/Tissue Inhibitor of Metalloproteinase 1 Expression in Murine Silicosis. Am. J. Respir. Crit. Care Med..

[191] Uchinami H., Seki E., Brenner D.A., D’Armiento J. (2006). Loss of MMP 13 Attenuates Murine Hepatic Injury and Fibrosis during Cholestasis. Hepatology.

[192] Knäuper V., Will H., López-Otin C., Smith B., Atkinson S.J., Stanton H., Hembry R.M., Murphy G. (1996). Cellular Mechanisms for Human Procollagenase-3 (MMP-13) Activation: Evidence that MT1-MMP (MMP-14) and gelatinase a (MMP-2) are able to generate active enzyme. J. Biol. Chem..

[193] Fallowfield J.A., Mizuno M., Kendall T.J., Constandinou C.M., Benyon R.C., Duffield J.S., Iredale J.P. (2007). Scar-Associated Macrophages Are a Major Source of Hepatic Matrix Metalloproteinase-13 and Facilitate the Resolution of Murine Hepatic Fibrosis. J. Immunol..

[194] García-Alvarez J., Ramirez R., Checa M., Nuttall R.K., Sampieri C.L., Edwards D.R., Selman M., Pardo A. (2009). Tissue Inhibitor of Metalloproteinase-3 Is up-Regulated by Transforming Growth Factor-Beta1 in Vitro and Expressed in Fibroblastic Foci in Vivo in Idiopathic Pulmonary Fibrosis. Exp. Lung Res..

[195] Cabrera S., Selman M., Lonzano-Bolaños A., Konishi K., Richards T.J., Kaminski N., Pardo A. (2013). Gene Expression Profiles Reveal Molecular Mechanisms Involved in the Progression and Resolution of Bleomycin-Induced Lung Fibrosis. Am. J. Physiol. Lung Cell. Mol. Physiol..

[196] Amar S., Smith L., Fields G.B. (2017). Matrix Metalloproteinase Collagenolysis in Health and Disease. Biochim. Biophys. Acta Mol. Cell Res..

[197] Rowe R.G., Keena D., Sabeh F., Willis A.L., Weiss S.J. (2011). Pulmonary Fibroblasts Mobilize the Membrane-Tethered Matrix Metalloprotease, MT1-MMP, to Destructively Remodel and Invade Interstitial Type I Collagen Barriers. Am. J. Physiol. Lung Cell. Mol. Physiol..

[198] Mu D., Cambier S., Fjellbirkeland L., Baron J.L., Munger J.S., Kawakatsu H., Sheppard D., Courtney Broaddus V., Nishimura S.L. (2002). The Integrin Avβ8 Mediates Epithelial Homeostasis through MT1-MMP–Dependent Activation of TGF-Β1. J. Cell Biol..

[199] Sabeh F., Ota I., Holmbeck K., Birkedal-Hansen H., Soloway P., Balbin M., Lopez-Otin C., Shapiro S., Inada M., Krane S. (2004). Tumor Cell Traffic through the Extracellular Matrix Is Controlled by the Membrane-Anchored Collagenase MT1-MMP. J. Cell Biol..

[200] Itoh Y. (2015). Membrane-Type Matrix Metalloproteinases: Their Functions and Regulations. Matrix Biol..

[201] Zigrino P., Brinckmann J., Niehoff A., Lu Y., Giebeler N., Eckes B., Kadler K.E., Mauch C. (2016). Fibroblast-Derived MMP-14 Regulates Collagen Homeostasis in Adult Skin. J. Investig. Dermatol..

[202] Mora A.L., Rojas M., Pardo A., Selman M. (2017). Emerging Therapies for Idiopathic Pulmonary Fibrosis, a Progressive Age-Related Disease. Nat. Rev. Drug Discov..

[203] Pendás A.M., Knäuper V., Puente X.S., Llano E., Mattei M.G., Apte S., Murphy G., López-Otín C. (1997). Identification and Characterization of a Novel Human Matrix Metalloproteinase with Unique Structural Characteristics, Chromosomal Location, and Tissue Distribution. J. Biol. Chem..

[204] Stracke J.O., Hutton M., Stewart M., Pendá A.M., Smith B., Ló Pez-Otin C., Murphy G., Knä V. (2000). Biochemical Characterization of the Catalytic Domain of Human Matrix Metalloproteinase 19. Evidence for a Role as a Potent Basement Membrane Degrading Enzyme. J. Biol. Chem..

[205] Suomela S., Kariniemi A.L., Impola U., Karvonen S.L., Snellman E., Uurasmaa T., Peltonen J., Saarialho-Kere U. (2003). Matrix Metalloproteinase-19 Is Expressed by Keratinocytes in Psoriasis. Acta Derm. Venereol..

[206] Zhu C.Q., Popova S.N., Brown E.R.S., Barsyte-Lovejoy D., Navab R., Shih W., Li M., Lu M., Jurisica I., Penn L.Z. (2007). Integrin Alpha 11 Regulates IGF2 Expression in Fibroblasts to Enhance Tumorigenicity of Human Non-Small-Cell Lung Cancer Cells. Proc. Natl. Acad. Sci. USA.

[207] Wilborn J., Crofford L.J., Burdick M.D., Kunkel S.L., Strieter R.M., Peters-Golden M. (1995). Cultured Lung Fibroblasts Isolated from Patients with Idiopathic Pulmonary Fibrosis Have a Diminished Capacity to Synthesize Prostaglandin E2 and to Express Cyclooxygenase-2. J. Clin. Investig..

[208] Mauch S., Kolb C., Kolb B., Sadowski T., Sedlacek R. (2002). Matrix Metalloproteinase-19 Is Expressed in Myeloid Cells in an Adhesion-Dependent Manner and Associates with the Cell Surface. J. Immunol..

[209] Gabasa M., Royo D., Molina-Molina M., Roca-Ferrer J., Pujols L., Picado C., Xaubet A., Pereda J. (2013). Lung Myofibroblasts Are Characterized by Down-Regulated Cyclooxygenase-2 and Its Main Metabolite, Prostaglandin E2. PLoS ONE.

[210] Beck I.M., Rückert R., Brandt K., Mueller M.S., Sadowski T., Brauer R., Schirmacher P., Mentlein R., Sedlacek R. (2008). MMP19 Is Essential for T Cell Development and T Cell-Mediated Cutaneous Immune Responses. PLoS ONE.

[211] Maldonado M., Salgado-Aguayo A., Herrera I., Cabrera S., Ortíz-Quintero B., Staab-Weijnitz C.A., Eickelberg O., Ramírez R., Manicone A.M., Selman M. (2018). Upregulation and Nuclear Location of MMP28 in Alveolar Epithelium of Idiopathic Pulmonary Fibrosis. Am. J. Respir. Cell Mol. Biol..

[212] Werner S.R., Mescher A.L., Neff A.W., King M.W., Chaturvedi S., Duffin K.L., Harty M.W., Smith R.C. (2007). Neural MMP-28 Expression Precedes Myelination during Development and Peripheral Nerve Repair. Dev. Dyn..

[213] Lohi J., Wilson C.L., Roby J.D., Parks W.C. (2001). Epilysin, a Novel Human Matrix Metalloproteinase (MMP-28) Expressed in Testis and Keratinocytes and in Response to Injury. J. Biol. Chem..

[214] Jian P., Yanfang T., Zhuan Z., Jian W., Xueming Z., Jian N. (2011). MMP28 (Epilysin) as a Novel Promoter of Invasion and Metastasis in Gastric Cancer. BMC Cancer.

[215] Momohara S., Okamoto H., Komiya K., Ikari K., Takeuchi M., Tomatsu T., Kamatani N., Clark I.M. (2004). Matrix Metalloproteinase 28/Epilysin Expression in Cartilage from Patients with Rheumatoid Arthritis and Osteoarthritis: Comment on the Article by Kevorkian et Al. Arthritis Rheum..

[216] Manicone A.M., Gharib S.A., Gong K.Q., Eddy W.E., Long M.E., Frevert C.W., Altemeier W.A., Parks W.C., Houghton A.M.G. (2017). Matrix Metalloproteinase-28 Is a Key Contributor to Emphysema Pathogenesis. Am. J. Pathol..

[217] Illman S.A., Lehti K., Keski-Oja J., Lohi J. (2006). Epilysin (MMP-28) Induces TGF-Beta Mediated Epithelial to Mesenchymal Transition in Lung Carcinoma Cells. J. Cell Sci..

[218] Song E., Ouyang N., Hörbelt M., Antus B., Wang M., Exton M.S. (2000). Influence of Alternatively and Classically Activated Macrophages on Fibrogenic Activities of Human Fibroblasts. Cell. Immunol..

[219] Chen L., Zhou Q., Xu B., Liu J., Shi L., Zhu D., Wu C., Jiang J. (2014). MT2-MMP Expression Associates with Tumor Progression and Angiogenesis in Human Lung Cancer. Int. J. Clin. Exp. Pathol..

[220] Ito E., Yana I., Fujita C., Irifune A., Takeda M., Madachi A., Mori S., Hamada Y., Kawaguchi N., Matsuura N. (2010). The Role of MT2-MMP in Cancer Progression. Biochem. Biophys. Res. Commun..

[221] Paye A., Truong A., Yip C., Cimino J., Blacher S., Munaut C., Cataldo D., Foidart J.M., Maquoi E., Collignon J. (2014). EGFR Activation and Signaling in Cancer Cells Are Enhanced by the Membrane-Bound Metalloprotease MT4-MMP. Cancer Res..

[222] Sun Q., Weber C.R., Sohail A., Bernardo M.M., Toth M., Zhao H., Turner J.R., Fridman R. (2007). MMP25 (MT6-MMP) Is Highly Expressed in Human Colon Cancer, Promotes Tumor Growth, and Exhibits Unique Biochemical Properties. J. Biol. Chem..

[223] Jung J.C., Wang P.X., Zhang G., Ezura Y., Fini M.E., Birk D.E. (2009). Collagen Fibril Growth during Chicken Tendon Development: Matrix Metalloproteinase-2 and Its Activation. Cell Tissue Res..

[224] Okimoto R.A., Breitenbuecher F., Olivas V.R., Wu W., Gini B., Hofree M., Asthana S., Hrustanovic G., Flanagan J., Tulpule A. (2017). Inactivation of Capicua Drives Cancer Metastasis. Nat. Genet..

[225] Shen Z., Wang X., Yu X., Zhang Y., Qin L. (2017). MMP16 Promotes Tumor Metastasis and Indicates Poor Prognosis in Hepatocellular Carcinoma. Oncotarget.

[226] Xie Y., Mustafa A., Yerzhan A., Merzhakupova D., Yerlan P., Orakov A.N., Wang X., Huang Y., Miao L. (2017). Nuclear Matrix Metalloproteinases: Functions Resemble the Evolution from the Intracellular to the Extracellular Compartment. Cell Death Discov..

[227] Zhang Y., Zhao H., Wang Y., Lin Y., Tan Y., Fang X., Zheng L. (2011). Non-Small Cell Lung Cancer Invasion and Metastasis Promoted by MMP-26. Mol. Med. Rep..

[228] Zhao Y.G., Xiao A.Z., Newcomer R.G., Park H.I., Kang T., Chung L.W.K., Swanson M.G., Zhau H.E., Kurhanewicz J., Sang Q.X.A. (2003). Activation of Pro-Gelatinase B by Endometase/Matrilysin-2 Promotes Invasion of Human Prostate Cancer Cells. J. Biol. Chem..

[229] Chaillan F.A., Rivera S., Marchetti E., Jourquin J., Werb Z., Soloway P.D., Khrestchatisky M., Roman F.S. (2006). Involvement of Tissue Inhibition of Metalloproteinases-1 in Learning and Memory in Mice. Behav. Brain Res..

[230] Caterina J.J., Yamada S., Caterina N.C.M., Longenecker G., Holmbäck K., Shi J., Yermovsky A.E., Engler J.A., Birkedal-Hansen H. (2000). Inactivating Mutation of the Mouse Tissue Inhibitor of Metalloproteinases-2(Timp-2) Gene Alters ProMMP-2 Activation. J. Biol. Chem..

[231] Koskivirta I., Kassiri Z., Rahkonen O., Kiviranta R., Oudit G.Y., McKee T.D., Kytö V., Saraste A., Jokinen E., Liu P.P. (2010). Mice with Tissue Inhibitor of Metalloproteinases 4 (Timp4) Deletion Succumb to Induced Myocardial Infarction but Not to Cardiac Pressure Overload. J. Biol. Chem..

[232] Leco K.J., Waterhouse P., Sanchez O.H., Gowing K.L.M., Poole A.R., Wakeham A., Mak T.W., Khokha R. (2001). Spontaneous Air Space Enlargement in the Lungs of Mice Lacking Tissue Inhibitor of Metalloproteinases-3 (TIMP-3). J. Clin. Investig..

[233] Kim K.H., Burkhart K., Chen P., Frevert C.W., Randolph-Habecker J., Hackman R.C., Soloway P.D., Madtes D.K. (2012). Tissue Inhibitor of Metalloproteinase-1 Deficiency Amplifies Acute Lung Injury in Bleomycin-Exposed Mice. Am. J. Respir. Cell Mol. Biol..

[234] Gill S.E., Huizar I., Bench E.M., Sussman S.W., Wang Y., Khokha R., Parks W.C. (2010). Tissue Inhibitor of Metalloproteinases 3 Regulates Resolution of Inflammation Following Acute Lung Injury. Am. J. Pathol..

[235] Tan S.Z., Liu C.H., Zhang W., Lu X., Ye W.C., Cai Z.Z., Liu P. (2006). Feature Changes of MMP-2/9 Activities and TIMP-1/2 Protein Expressions during the Progression of Pulmonary Fibrosis in Rats. Zhong Xi Yi Jie He Xue Bao.

[236] Zhou X.M., Wang G.L., Wang X.B., Liu L., Zhang Q., Yin Y., Wang Q.Y., Kang J., Hou G. (2017). GHK Peptide Inhibits Bleomycin-Induced Pulmonary Fibrosis in Mice by Suppressing TGFβ1/Smad-Mediated Epithelial-to-Mesenchymal Transition. Front. Pharmacol..

[237] Zuo W., Zhao J., Huang J., Zhou W., Lei Z., Huang Y., Huang Y., Li H. (2017). Effect of Bosentan Is Correlated with MMP-9/TIMP-1 Ratio in Bleomycin-induced Pulmonary Fibrosis. Biomed. Rep..

[238] Zhou Y., He Z., Gao Y., Zheng R., Zhang X., Zhao L., Tan M. (2016). Induced Pluripotent Stem Cells Inhibit Bleomycin-Induced Pulmonary Fibrosis in Mice through Suppressing TGF-Β1/Smad-Mediated Epithelial to Mesenchymal Transition. Front. Pharmacol..

[239] Madtes D.K., Elston A.L., Kaback L.A., Clark J.G. (2012). Selective Induction of Tissue Inhibitor of Metalloproteinase-1 in Bleomycin-Induced Pulmonary Fibrosis. Am. J. Respir. Cell Mol. Biol..

[240] Ramos C., Montaño M., García-Alvarez J., Ruiz V., Uhal B.D., Selman M., Pardo A. (2001). Fibroblasts from Idiopathic Pulmonary Fibrosis and Normal Lungs Differ in Growth Rate, Apoptosis, and Tissue Inhibitor of Metalloproteinases Expression. Am. J. Respir. Cell Mol. Biol..

[241] Pardo A., Selman M., Ramirez R., Ramos C., Montano M., Stricklin G., Raghu G. (1992). Production of Collagenase and Tissue Inhibitor of Metalloproteinases by Fibroblasts Derived from Normal and Fibrotic Human Lungs. Chest.

[242] Hayashi T., Stetler-Stevenson W.G., Fleming M.V., Fishback N., Koss M.N., Liotta L.A., Ferrans V.J., Travis W.D. (1996). Immunohistochemical Study of Metalloproteinases and Their Tissue Inhibitors in the Lungs of Patients with Diffuse Alveolar Damage and Idiopathic Pulmonary Fibrosis. Am. J. Pathol..

[243] Menou A., Duitman J.W., Crestani B. (2018). The Impaired Proteases and Anti-Proteases Balance in Idiopathic Pulmonary Fibrosis. Matrix Biol..

[244] Miyauchi T., Kanekura T., Yamaoka A., Ozawa M., Miyazawa S., Muramatasu T. (1990). Basigin, a New, Broadly Distributed Member of the Immunoglobulin Superfamily, Has Strong Homology with Both the Immunoglobulin V Domain and the β-Chain of Major Histocompatibility Complex Class II Antigen. J. Biochem..

[245] Biswas C. (1984). Collagenase Stimulation in Cocultures of Human Fibroblasts and Human Tumor Cells. Cancer Lett..

[246] Biswas C., Zhang Y., DeCastro R., Guo H., Nakamura T., Kataoka H., Nabeshima K. (1995). The Human Tumor Cell-Derived Collagenase Stimulatory Factor (Renamed EMMPRIN) Is a Member of the Immunoglobulin Superfamily. Cancer Res..

[247] Kaname T., Miyauchi T., Kuwano A., Matsuda Y., Muramatsu T., Kajii T. (1993). Mapping Basigin (BSG), a Member of the Immunoglobulin Superfamily, to 19p13.3. Cytogenet. Genome Res..

[248] Grass G.D., Toole B.P. (2015). How, with Whom and When: An Overview of CD147-Mediated Regulatory Networks Influencing Matrix Metalloproteinase Activity. Biosci. Rep..

[249] Kumar D., Vetrivel U., Parameswaran S., Subramanian K.K. (2019). Structural Insights on Druggable Hotspots in CD147: A Bull’s Eye View. Life Sci..

[250] Sameshima T., Nabeshima K., Toole B.P., Yokogami K., Okada Y., Goya T., Koono M., Wakisaka S. (2000). Glioma Cell Extracellular Matrix Metalloproteinase Inducer (EMMPRIN) (CD147) Stimulates Production of Membrane-Type Matrix Metalloproteinases and Activated Gelatinase A in Co-Cultures with Brain-Derived Fibroblasts. Cancer Lett..

[251] Yang N., Higuchi O., Ohashi K., Nagata K., Wada A., Kangawa K., Nishida E., Mizuno K. (1998). Cofilin Phosphorylation by LIM-Kinase 1 and Its Role in Rac-Mediated Actin Reorganization. Nature.

[252] Guindolet D., Gabison E.E. (2020). Role of CD147 (EMMPRIN/Basigin) in Tissue Remodeling. Anat. Rec..

[253] Liao C.-G., Kong L.-M., Song F., Xing J.-L., Wang L.-X., Sun Z.-J., Tang H., Yao H., Zhang Y., Wang L. (2011). Characterization of Basigin Isoforms and the Inhibitory Function of Basigin-3 in Human Hepatocellular Carcinoma Proliferation and Invasion. Mol. Cell. Biol..

[254] Gabison E.E., Hoang-Xuan T., Mauviel A., Menashi S. (2005). EMMPRIN/CD147, an MMP Modulator in Cancer, Development and Tissue Repair. Biochimie.

[255] Yan L., Zucker S., Toole B.P. (2005). Roles of the Multifunctional Glycoprotein, Emmprin (Basigin; CD147), in Tumour Progression. Thromb. Haemost..

[256] Yu X.L., Hu T., Du J.M., Ding J.P., Yang X.M., Zhang J., Yang B., Shen X., Zhang Z., Zhong W.D. (2008). Crystal Structure of HAb18G/CD147: Implications for Immunoglobulin Superfamily Homophilic Adhesion. J. Biol. Chem..

[257] Li J.H., Huang W., Lin P., Wu B., Fu Z.G., Shen H.M., Jing L., Liu Z.Y., Zhou Y., Meng Y. (2016). N-Linked Glycosylation at Asn152 on CD147 Affects Protein Folding and Stability: Promoting Tumour Metastasis in Hepatocellular Carcinoma. Sci. Rep..

[258] Papadimitropoulou A., Mamalaki A. (2013). The Glycosylated IgII Extracellular Domain of EMMPRIN Is Implicated in the Induction of MMP-2. Mol. Cell. Biochem..

[259] Tang W., Chang S.B., Hemler M.E. (2004). Links between CD147 Function, Glycosylation, and Caveolin-1. Mol. Biol. Cell.

[260] Chow A.K., Cena J., El-Yazbi A.F., Crawford B.D., Holt A., Cho W.J., Daniel E.E., Schulz R. (2007). Caveolin-1 Inhibits Matrix Metalloproteinase-2 Activity in the Heart. J. Mol. Cell. Cardiol..

[261] Belton R.J., Chen L., Mesquita F.S., Nowak R.A. (2008). Basigin-2 Is a Cell Surface Receptor for Soluble Basigin Ligand. J. Biol. Chem..

[262] Daniel Grass G., Bratoeva M., Toole B.P. (2012). Regulation of Invadopodia Formation and Activity by CD147. J. Cell Sci..

[263] Knutti N., Kuepper M., Friedrich K. (2015). Soluble Extracellular Matrix Metalloproteinase Inducer (EMMPRIN, EMN) Regulates Cancer-Related Cellular Functions by Homotypic Interactions with Surface CD147. FEBS J..

[264] Von Ungern-Sternberg S.N.I., Zernecke A., Seizer P. (2018). Extracellular Matrix Metalloproteinase Inducer EMMPRIN (CD147) in Cardiovascular Disease. Int. J. Mol. Sci..

[265] Pinheiro C., Longatto-Filho A., Simões K., Jacob C.E., Bresciani C.J.C., Zilberstein B., Cecconello I., Alves V.A.F., Schmitt F., Baltazar F. (2009). The Prognostic Value of CD147/EMMPRIN Is Associated with Monocarboxylate Transporter 1 Co-Expression in Gastric Cancer. Eur. J. Cancer.

[266] Toole B.P. (2020). The CD147-HYALURONAN Axis in Cancer. Anat. Rec..

[267] Yurchenko V., Constant S., Bukrinsky M. (2006). Dealing with the Family: CD147 Interactions with Cyclophilins. Immunology.

[268] Takahashi M., Suzuki S., Ishikawa K. (2012). Cyclophilin A-EMMPRIN Interaction Induces Invasion of Head and Neck Squamous Cell Carcinoma. Oncol. Rep..

[269] Li M., Zhai Q., Bharadwaj U., Wang M., Li F., Fisher W.E., Chen C., Yao Q. (2006). Cyclophilin A Is Overexpressed in Human Pancreatic Cancer Cells and Stimulates Cell Proliferation through CD147. Cancer.

[270] Pushkarsky T., Yurchenko V., Vanpouille C., Brichacek B., Vaisman I., Hatakeyama S., Nakayama K.I., Sherry B., Bukrinsky M.I. (2005). Cell Surface Expression of CD147/EMMPRIN Is Regulated by Cyclophilin 60. J. Biol. Chem..

[271] Li Y., Wu J., Song F., Tang J., Wang S.J., Yu X.L., Chen Z.N., Jiang J.L. (2012). Extracellular Membrane-Proximal Domain of HAb18G/CD147 Binds to Metal Ion-Dependent Adhesion Site (MIDAS) Motif of Integrin Β1 to Modulate Malignant Properties of Hepatoma Cells. J. Biol. Chem..

[272] Khayati F., Pérez-Cano L., Maouche K., Sadoux A., Boutalbi Z., Podgorniak M.-P., Maskos U., Setterblad N., Janin A., Calvo F. (2015). EMMPRIN/CD147 Is a Novel Coreceptor of VEGFR-2 Mediating Its Activation by VEGF. Oncotarget.

[273] Zhang W., Zhao P., Xu X.L., Cai L., Song Z.S., Cao D.Y., Tao K.S., Zhou W.P., Chen Z.N., Dou K.F. (2013). Annexin A2 Promotes the Migration and Invasion of Human Hepatocellular Carcinoma Cells In Vitro by Regulating the Shedding of CD147-Harboring Microvesicles from Tumor Cells. PLoS ONE.

[274] Priglinger C.S., Szober C.M., Priglinger S.G., Merl J., Euler K.N., Kernt M., Gondi G., Behler J., Geerlof A., Kampik A. (2013). Galectin-3 Induces Clustering of CD147 and Integrin-Β1 Transmembrane Glycoprotein Receptors on the RPE Cell Surface. PLoS ONE.

[275] Luo Z., Zhang X., Zeng W., Su J., Yang K., Lu L., Lim C.B., Tang W., Wu L., Zhao S. (2016). TRAF6 Regulates Melanoma Invasion and Metastasis through Ubiquitination of Basigin. Oncotarget.

[276] Zhou S., Liao L., Chen C., Zeng W., Liu S., Su J., Zhao S., Chen M., Kuang Y., Chen X. (2013). CD147 Mediates Chemoresistance in Breast Cancer via ABCG2 by Affecting Its Cellular Localization and Dimerization. Cancer Lett..

[277] Kong L.M., Liao C.G., Fei F., Guo X., Xing J.L., Chen Z.N. (2010). Transcription Factor Sp1 Regulates Expression of Cancer-Associated Molecule CD147 in Human Lung Cancer. Cancer Sci..

[278] Polette M., Gilles C., Marchand V., Lorenzato M., Toole B., Tournier J.M., Zucker S., Birembaut P. (1997). Tumor Collagenase Stimulatory Factor (TCSF) Expression and Localization in Human Lung and Breast Cancers. J. Histochem. Cytochem..

[279] Caudroy S., Polette M., Tournier J.M., Burlet H., Toole B., Zucker S., Birembaut P. (1999). Expression of the Extracellular Matrix Metalloproteinase Inducer (EMMPRIN) and the Matrix Metalloproteinase-2 in Bronchopulmonary and Breast Lesions. J. Histochem. Cytochem..

[280] Suzuki S., Sato M., Senoo H., Ishikawa K. (2004). Direct Cell-Cell Interaction Enhances pro-MMP-2 Production and Activation in Co-Culture of Laryngeal Cancer Cells and Fibroblasts: Involvement of EMMPRIN and MT1-MMP. Exp. Cell Res..

[281] Taylor P.M., Woodfield R.J., Hodgkin M.N., Pettitt T.R., Martin A., Kerr D.J., Wakelam M.J.O. (2002). Breast Cancer Cell-Derived EMMPRIN Stimulates Fibroblast MMP2 Release through a Phospholipase A(2) and 5-Lipoxygenase Catalyzed Pathway. Oncogene.

[282] Nabeshima K., Suzumiya J., Nagano M., Ohshima K., Toole B.P., Tamura K., Iwasaki H., Kikuchi M. (2004). Emmprin, a Cell Surface Inducer of Matrix Metalloproteinases (MMPs), Is Expressed in T-Cell Lymphomas. J. Pathol..

[283] Jiang J.L., Zhou Q., Yu M.K., Ho L.S., Chen Z.N., Chan H.C. (2001). The Involvement of HAb18G/CD147 in Regulation of Store-Operated Calcium Entry and Metastasis of Human Hepatoma Cells. J. Biol. Chem..

[284] Van Den Oord J.J., Paemen L., Opdenakker G., De Wolf-Peeters C. (1997). Expression of Gelatinase B and the Extracellular Matrix Metalloproteinase Inducer EMMPRIN in Benign and Malignant Pigment Cell Lesions of the Skin. Am. J. Pathol..

[285] Kim H.S., Kim H.J., Lee M.R., Han I. (2021). EMMPRIN Expression Is Associated with Metastatic Progression in Osteosarcoma. BMC Cancer.

[286] Savarese-Brenner B., Heugl M., Rath B., Schweizer C., Obermayr E., Stickler S., Hamilton G. (2022). MUC1 and CD147 Are Promising Markers for the Detection of Circulating Tumor Cells in Small Cell Lung Cancer. Anticancer Res..

[287] Betsuyaku T., Tanino M., Nagai K., Nasuhara Y., Nishimura M., Senior R.M. (2012). Extracellular Matrix Metalloproteinase Inducer Is Increased in Smokers’ Bronchoalveolar Lavage Fluid. Am. J. Respir. Crit. Care Med..

[288] Major T.C., Liang L., Lu X., Rosebury W., Bocan T.M.A. (2002). Extracellular Matrix Metalloproteinase Inducer (EMMPRIN) Is Induced upon Monocyte Differentiation and Is Expressed in Human Atheroma. Arterioscler. Thromb. Vasc. Biol..

[289] Foda H.D., Rollo E.E., Drews M., Conner C., Appelt K., Shalinsky D.R., Zucker S. (2001). Ventilator-Induced Lung Injury Upregulates and Activates Gelatinases and EMMPRIN: Attenuation by the Synthetic Matrix Metalloproteinase Inhibitor, Prinomastat (AG3340). Am. J. Respir. Cell Mol. Biol..

[290] Spinale F.G., Coker M.L., Heung L.J., Bond B.R., Gunasinghe H.R., Etoh T., Goldberg A.T., Zellner J.L., Crumbley A.J. (2000). A Matrix Metalloproteinase Induction/Activation System Exists in the Human Left Ventricular Myocardium and Is Upregulated in Heart Failure. Circulation.

[291] Fan Q.W., Kadomatsu K., Uchimura K., Muramatsu T. (1998). Embigin/Basigin Subgroup of the Immunoglobulin Superfamily: Different Modes of Expression during Mouse Embryogenesis and Correlated Expression with Carbohydrate Antigenic Markers. Dev. Growth Differ..

[292] Chen X., Kanekura T., Kanzaki T. (2001). Expression of Basigin in Human Fetal, Infantile and Adult Skin and in Basal Cell Carcinoma. J. Cutan. Pathol..

[293] Igakura T., Kadomatsu K., Kaname T., Muramatsu H., Fan Q.W., Miyauchi T., Toyama Y., Kuno N., Yuasa S., Takahashi M. (1998). A Null Mutation in Basigin, an Immunoglobulin Superfamily Member, Indicates Its Important Roles in Peri-Implantation Development and Spermatogenesis. Dev. Biol..

[294] Bremnes R.M., Dønnem T., Al-Saad S., Al-Shibli K., Andersen S., Sirera R., Camps C., Marinez I., Busund L.T. (2011). The Role of Tumor Stroma in Cancer Progression and Prognosis: Emphasis on Carcinoma-Associated Fibroblasts and Non-Small Cell Lung Cancer. J. Thorac. Oncol..

[295] Li H.Y., Ju D., Zhang D.W., Li H., Kong L.M., Guo Y., Li C., Wang X.L., Chen Z.N., Bian H. (2015). Activation of TGF-Β1-CD147 Positive Feedback Loop in Hepatic Stellate Cells Promotes Liver Fibrosis. Sci. Rep..

[296] Xu J., Lu Y., Qiu S., Chen Z.N., Fan Z. (2013). A Novel Role of EMMPRIN/CD147 in Transformation of Quiescent Fibroblasts to Cancer-Associated Fibroblasts by Breast Cancer Cells. Cancer Lett..

[297] Guillot S., Delaval P., Brinchault G., Caulet-Maugendre S., Depince A., Lena H., Delatour B., Lagente V., Martin-Chouly C. (2006). Increased Extracellular Matrix Metalloproteinase Inducer (EMMPRIN) Expression in Pulmonary Fibrosis. Exp. Lung Res..

[298] Betsuyaku T., Kadomatsu K., Griffin G.L., Muramatsu T., Senior R.M. (2003). Increased Basigin in Bleomycin-Induced Lung Injury. Am. J. Respir. Cell Mol. Biol..

[299] Hasaneen N.A., Cao J., Pulkoski-Gross A., Zucker S., Foda H.D. (2016). Extracellular Matrix Metalloproteinase Inducer (EMMPRIN) Promotes Lung Fibroblast Proliferation, Survival and Differentiation to Myofibroblasts. Respir. Res..

[300] Huet E., Vallée B., Szul D., Verrecchia F., Mourah S., Jester J.V., Hoang-Xuan T., Menashi S., GaMson E.E. (2008). Extracellular Matrix Metalloproteinase Inducer/CD147 Promotes Myofibroblast Differentiation by Inducing Alpha-Smooth Muscle Actin Expression and Collagen Gel Contraction: Implications in Tissue Remodeling. FASEB J..

[301] Woods E.L., Grigorieva I.V., Midgley A.C., Brown C.V.M., Lu Y.A., Phillips A.O., Bowen T., Meran S., Steadman R. (2021). CD147 Mediates the CD44s-Dependent Differentiation of Myofibroblasts Driven by Transforming Growth Factor-β 1. J. Biol. Chem..

[302] Wu X.D., Zhang M.Y., Chen Y.T., Yao H., Zhang Q., Wang W.J., Fu D.F., Wei R.J., Zhang J.Y., Li Y. (2019). Generation and Characterization of Fibroblast-Specific Basigin Knockout Mice. Mol. Biotechnol..

[303] Geng J.-j., Zhang K., Chen L.-n., Miao J.-l., Yao M., Ren Y., Fu Z.-g., Chen Z.-n., Zhu P. (2014). Enhancement of CD147 on M1 Macrophages Induces Differentiation of Th17 Cells in the Lung Interstitial Fibrosis. Biochim. Biophys. Acta.

[304] Barth K., Bläsche R., Kasper M. (2006). Lack of Evidence for Caveolin-1 and CD147 Interaction before and after Bleomycin-Induced Lung Injury. Histochem. Cell Biol..

[305] Liu F., Yu F., Lu Y.Z., Cheng P.P., Liang L.M., Wang M., Chen S.J., Huang Y., Song L.J., He X.L. (2020). Crosstalk between Pleural Mesothelial Cell and Lung Fibroblast Contributes to Pulmonary Fibrosis. Biochim. Biophys. Acta Mol. Cell Res..

